# Revisiting promyelocytic leukemia protein targeting by human cytomegalovirus immediate-early protein 1

**DOI:** 10.1371/journal.ppat.1008537

**Published:** 2020-05-04

**Authors:** Christina Paulus, Thomas Harwardt, Bernadette Walter, Andrea Marxreiter, Marion Zenger, Edith Reuschel, Michael M. Nevels

**Affiliations:** 1 Biomedical Sciences Research Complex, University of St Andrews, St Andrews, United Kingdom; 2 Institute for Medical Microbiology and Hygiene, University of Regensburg, Regensburg, Germany; 3 Department of Obstetrics and Gynecology, Clinic St. Hedwig at Hospital Barmherzige Brüder Regensburg, Regensburg, Germany; State University of New York Upstate Medical University, UNITED STATES

## Abstract

Promyelocytic leukemia (PML) bodies are nuclear organelles implicated in intrinsic and innate antiviral defense. The eponymous PML proteins, central to the self-organization of PML bodies, and other restriction factors found in these organelles are common targets of viral antagonism. The 72-kDa immediate-early protein 1 (IE1) is the principal antagonist of PML bodies encoded by the human cytomegalovirus (hCMV). IE1 is believed to disrupt PML bodies by inhibiting PML SUMOylation, while PML was proposed to act as an E3 ligase for IE1 SUMOylation. PML targeting by IE1 is considered to be crucial for hCMV replication at low multiplicities of infection, in part via counteracting antiviral gene induction linked to the cellular interferon (IFN) response. However, current concepts of IE1-PML interaction are largely derived from mutant IE1 proteins known or predicted to be metabolically unstable and globally misfolded. We performed systematic clustered charge-to-alanine scanning mutagenesis and identified a stable IE1 mutant protein (IE1cc172-176) with wild-type characteristics except for neither interacting with PML proteins nor inhibiting PML SUMOylation. Consequently, IE1cc172-176 does not associate with PML bodies and is selectively impaired for disrupting these organelles. Surprisingly, functional analysis of IE1cc172-176 revealed that the protein is hypermodified by mixed SUMO chains and that IE1 SUMOylation depends on nucleosome rather than PML binding. Furthermore, a mutant hCMV expressing IE1cc172-176 was only slightly attenuated compared to an IE1-null virus even at low multiplicities of infection. Finally, hCMV-induced expression of cytokine and IFN-stimulated genes turned out to be reduced rather than increased in the presence of IE1cc172-176 relative to wild-type IE1. Our findings challenge present views on the relationship of IE1 with PML and the role of PML in hCMV replication. This study also provides initial evidence for the idea that disruption of PML bodies upon viral infection is linked to activation rather than inhibition of innate immunity.

## Author summary

Promyelocytic leukemia (PML) bodies are liquid droplet-like structures organized by the eponymous PML proteins in the nuclei of our cells. PML bodies have been implicated in the antiviral host cell response to infection. Consequently, viruses have evolved mechanisms that target the proteins composing PML bodies. Immediate-early protein 1 (IE1) is considered the principal antagonist of PML bodies produced by the human cytomegalovirus, one of eight human herpesviruses. Previous work suggested that the interaction between IE1 and PML and the consequent disruption of PML bodies serves a critical role in viral replication by counteracting the cellular antiviral response. However, this picture has emerged largely from studying mutant IE1 proteins known or predicted to be unstable. We systematically screened for stable IE1 variants and identified a mutant protein selectively defective for PML interaction. Unexpectedly, the IE1 mutant supported viral replication almost as efficiently as the wild-type protein. Moreover, lower instead of higher (as expected) levels of antiviral gene expression were observed with the mutant compared to the wild-type. These results suggest that disruption of PML bodies is linked to the induction rather than inhibition of antiviral gene expression. Our findings challenge current views regarding the role of PML bodies in viral infection.

## Introduction

Promyelocytic leukemia (PML) bodies, also known as nuclear domain 10, are membrane-less nuclear organelles present in most cells (reviewed in [1, 2]). PML bodies are heterogeneous and dynamic, ranging in size between 0.1 and 1.0 μm and typically displaying as five to 30 spherical structures per nucleus interspersed between chromatin. They are composed of the eponymous PML proteins, also known as tripartite motif (TRIM) 19, that self-organize into a shell-like scaffold. The PML scaffold forms, in part by phase separation, around an inner core which sometimes contains nucleic acids [3–10]. Embedded in the scaffold or core are numerous unrelated proteins, most of which associate with PML bodies in a conditional and transient manner [11, 12]. Proteins constitutively residing in PML bodies include the six nuclear PML isoforms, the speckled 100 kDa (Sp100) nuclear antigens, the death domain-associated protein (Daxx) and the small ubiquitin-like modifier (SUMO) family members SUMO1, SUMO2, SUMO3 and SUMO5 [13, 14]. PML bodies are considered to be SUMOylation hubs, and most proteins associated with these organelles are post-translationally modified by one or more SUMO paralogs [12, 15]. While SUMO1 attaches only as a monomer, SUMO2, SUMO3 and SUMO5 can form polymeric chains on target proteins including PML [14, 16]. The biogenesis and integrity of functional PML bodies depends on oligomerization and poly-SUMOylation of PML proteins at three major lysine residues. In the absence of SUMOylation, PML proteins may condense into spherical structures unable to recruit other proteins [17, 18]. For instance, aggregates referred to as mitotic accumulations of PML protein (MAPPs) are known to form following PML de-SUMOylation and breakdown of PML bodies at the onset of mitosis [19–23].

Although any unifying biochemical function of PML bodies remains to be established, they have been involved in a wide variety of biological processes (reviewed in [1, 24]). Some of these processes relate to intrinsic immunity, the first intracellular line of defense against invading pathogens (reviewed in [25, 26]). PML bodies may confer intrinsic immunity as a whole by entrapping viral genomes or capsids [8–10, 27, 28]. In addition, PML, Sp100, Daxx and other proteins associated with PML bodies act individually as restriction factors for numerous RNA and DNA viruses by several mechanisms including transcriptional repression (reviewed in [26, 29]). Moreover, PML has been identified as a key regulator of cytokine responses and innate immunity (reviewed in [29, 30]). Certain nuclear PML isoforms are positive regulators of interferon (IFN) synthesis. In addition, PML proteins may directly promote induction of some IFN-stimulated genes (ISGs) triggered by IFNβ or IFNγ [29, 31–34]. Conversely, the PML gene and several other genes encoding constituents of PML bodies are bona fide ISGs [35, 36]. More broadly, PML appears to facilitate innate immunity and inflammation by affecting expression of cytokines beyond IFNs including tumor necrosis factor (TNF) and C-C motif chemokine ligand 5 (CCL5) [37–39]. The mechanisms underlying positive regulation of cytokine expression and signaling by PML have not been fully elucidated. However, PML was shown to associate with transcription factors that control IFN and ISG expression and to facilitate their assembly on target gene promoters [32, 34, 40]. These findings demonstrate a key role of PML in antiviral restriction as well as cytokine-induced antiviral and inflammatory states.

Unsurprisingly, many viruses have evolved mechanisms to inactivate the antiviral properties associated with PML bodies and their restriction factors. Besides direct and sometimes mutual effects between the cellular factors and viral antagonists, PML targeting usually leads to structural changes in PML bodies or even a complete loss of organelle integrity. Disruption of PML bodies is widely regarded as a mechanism by which viruses antagonize the intrinsic and innate immune responses ascribed to these organelles or their proteins (reviewed in [24, 41]). One of the best-studied viral ‘offenders’ of PML bodies is the immediate-early protein 1 (IE1) encoded by the human cytomegalovirus (hCMV), an opportunistic pathogen of the herpesvirus family (reviewed in [42, 43]).

The hCMV IE1 (UL123) and IE2 (UL122) proteins are translated from alternatively spliced and polyadenylated mRNAs originating from the major immediate-early transcription unit. They are the first viral gene products newly synthesized upon infection (reviewed in [43, 44]). The main IE1 isoform (herein referred to as IE1) appears as a 72-kDa species in protein gels and is composed of four structurally and functionally distinct regions. A short N-terminal domain (amino acids 1 to 24) is predicted to be intrinsically disordered and contains one of at least two nuclear localization signals [45–49]. The central region downstream from the N-terminal part has been termed the core domain (amino acids 25 to 378). The core domain of the IE1 ortholog from Rhesus cytomegalovirus, predicted to be conserved in hCMV, exhibits a femur-like fold composed of 11 α-helices resembling the coiled-coil domain of TRIM family members. This domain mediates binding to PML and other TRIM proteins as well as homo-dimer formation [49, 50]. Beyond the core domain lies a region frequently referred to as ‘acidic domain’ (amino acids 379 to 475). The acidic domain is believed to be intrinsically disordered and contains four low complexity motifs termed acidic domain 1 (AD1), serine-proline-rich (S/P), AD2 and AD3 based on their compositional bias [45, 51, 52]. Embedded between AD1 and S/P is a sequence (amino acids 410 to 420) that serves as a binding site for at least two members of the signal transducer and activator of transcription (STAT) family of proteins, STAT2 and STAT3 [45, 52, 53]. At lysine 450, located between AD2 and AD3, IE1 can undergo conjugation to SUMO1 or SUMO3 [54–56]. It has been suggested that PML serves as an E3 ligase for SUMO modification of IE1, although protein inhibitor of activated STAT 1 (PIAS1) appears to enhance IE1 SUMOylation as well [57, 58]. Finally, C-terminal amino acids 476 to 491 represent the chromatin tethering domain (CTD) [48, 59]. This domain contains a nucleosome binding motif (NBM) that targets the acidic patch formed by histones H2A and H2B on the nucleosome surface and thereby mediates association with both interphase and mitotic chromatin [60–62]. The CTD peptide adopts an extended, v-shaped conformation with a short α-helix at its C-terminus and has been implicated in regulation of higher-order chromatin structure and viral genome maintenance during hCMV latency [62, 63]. In the context of high passage hCMV strains, IE1 has been shown to be required for efficient viral replication in fibroblasts under conditions of low but not high multiplicity of infection (MOI) [64–66]. However, low passage hCMV strains deficient in IE1 are substantially attenuated at both low and high MOI in fibroblasts suggesting that the protein serves an even more important role in viral replication than originally thought ([67] and this work).

Initially characterized as a promiscuous activator of transcription, IE1 has later emerged as an antagonist of intrinsic and innate immune responses to hCMV infection (reviewed in [43, 68]). At least two distinct activities contribute to evasion of intrinsic and innate immunity by IE1. First, the STAT binding motif in the viral protein confers complex formation with STAT2 [45, 52, 53, 69]. The IE1-STAT2 interaction results in diminished DNA binding of IFN-stimulated gene factor 3, a trimeric complex of STAT1, STAT2 and IFN regulatory factor 9 required for induction of type I ISGs many of which encode antiviral products. Consequently, IE1 inhibits type I ISG activation conferring relative resistance to the antiviral activities of IFNα and IFNβ on hCMV [45, 53, 69]. In addition, the STAT binding motif mediates interaction between IE1 and STAT3 resulting in decreased interleukin 6 (IL6)-induced gene expression and increased expression of type II ISGs linked to STAT1 phosphorylation [52, 70–72]. Secondly, the IE1 core domain interacts with the coiled-coil domain of PML [49, 73]. Complex formation between IE1 and PML results in transient co-localization of IE1 at PML bodies. This interaction is followed by inhibited oligomerization of PML, reduced *de novo* PML SUMOylation and disruption of PML bodies [56, 74–76]. PML targeting by IE1 is thought to promote hCMV replication in at least two ways: by relieving viral transcription from repression mediated by PML proteins or PML bodies (intrinsic immunity) and by inhibiting PML-dependent IFN and ISG expression triggered by viral infection (innate immunity) [34, 74, 77, 78]. Besides PML isoforms and SUMO paralogs, IE1 has also been shown to target two other resident proteins of PML bodies, Daxx and Sp100A [51, 79, 80], most likely to antagonize antiviral restriction via transcriptional repression. While the IE1-Daxx interaction has not been studied in detail, the viral protein was shown to reduce the SUMOylation of Sp100A and to target the cellular protein for proteasomal degradation [51, 79].

Numerous studies have investigated how IE1-PML interaction and disruption of PML bodies affect the hCMV productive cycle (reviewed in [42, 81]). It has been concluded that PML targeting is a central activity by which IE1 antagonizes intrinsic and innate immunity to facilitate hCMV replication [34, 77, 78, 82]. However, most of these studies were merely of correlative nature and involved IE1 mutants with disrupted core domains resulting in proteins known to be metabolically unstable and predicted to be globally misfolded. For example, many conclusions have relied on IE1 mutant proteins and viruses that replace leucine 174 with proline (L174P) [56, 77, 83, 84]. To our knowledge, a mutation in IE1 that produces a metabolically stable protein and selectively abolishes PML binding without affecting other activities has not been identified. Here we performed systematic clustered charge-to-alanine scanning of IE1 to identify a mutant protein (IE1cc172-176) that fails to interact physically with PML, to inhibit PML SUMOylation and to associate with PML bodies. However, IE1cc172-176 accumulates to wild-type levels in host cell nuclei, and functions unrelated to PML are not affected by the mutation. We utilized IE1cc172-176 to re-evaluate the contribution of PML targeting by IE1 to hCMV replication. Our findings challenge the predominant view that interaction with PML is central to the function of IE1 and provide evidence for antiviral rather than proviral effects of PML body disruption.

## Results

### Clustered charge-to-alanine scanning of IE1 identifies stable core domain mutants

**Fig 1:**
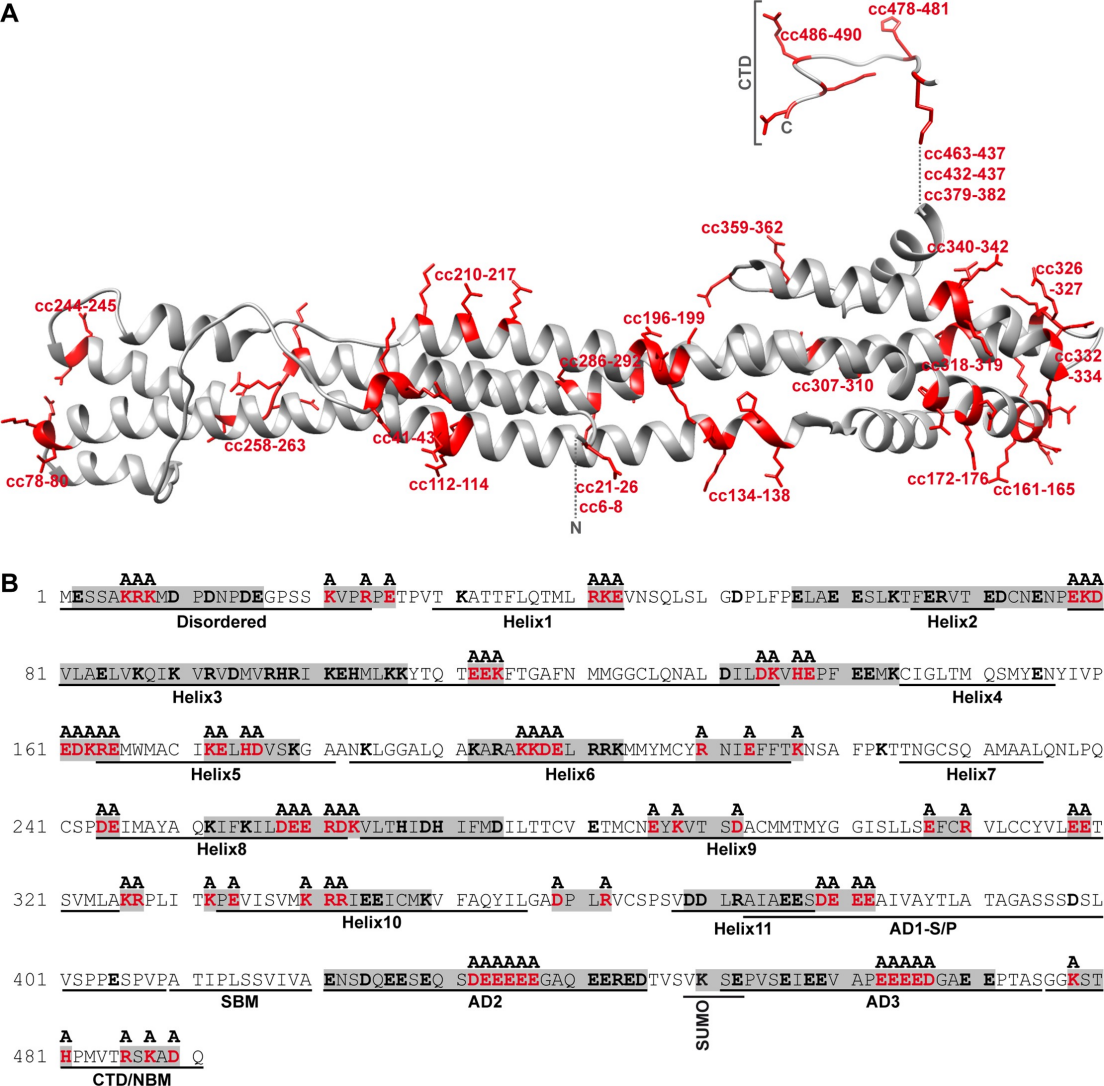
Design of clustered charge-to-alanine IE1 mutants. (A) Tertiary protein structure of hCMV IE1 showing the core domain modelled on the orthologous domain of rhCMV IE1 (PDB 4WID) [49] using Phyre2 and the C-terminal CTD (PDB 5E5A) [62]. The three-dimensional structures of the N-terminal domain and the ‘acidic domain’ (both replaced by dotted lines) have not been determined but are predicted to be disordered. Residues substituted with alanine in the indicated 24 clustered charge (‘cc’) mutants are shown in red. (B) Primary protein structure of hCMV IE1 showing charged residues (bold) and the 24 clustered charges (gray boxes). Residues substituted with alanine in the clustered charge mutants are shown in red. The presumably disordered N-terminal domain, the 11 α-helices (predicted based on rhCMV IE1) [49] composing the core domain, the low complexity motifs (AD1, S/P, AD2 and AD3) [45] of the presumably disordered ‘acidic domain’, the STAT binding motif (SBM) [52] and the CTD [59] including the NBM [92] are indicated. The SUMOylation motif and SUMO attachment site (lysine 450) are marked as well.

To identify stable mutant proteins that allow for conclusive discrimination between individual activities of IE1, we performed systematic clustered charge-to-alanine mutagenesis across the whole length of the viral protein. Charged residues clustered in the primary sequence are likely to be located on the surface of the folded protein where they may facilitate interactions with other proteins. Conversely, the replacement of clustered charged residues with alanine may disrupt such interactions without significantly affecting the three-dimensional structure and stability of the protein [85–90]. A clustered charge is commonly defined as two or more charged residues in a window of five amino acids [87, 91]. According to this definition, 24 clustered charges were identified across the 491 amino acids of IE1 (Fig 1). Two to six charged residues within each cluster, selected based on charge density, were changed to alanine by site-directed mutagenesis (Fig 1B). The resulting 24 clustered charge mutants are referred to as IE1cc6-8, IE1cc21-26, etc with numbers indicating the residues N- and C-terminal of the target site. As expected, most residues targeted by clustered charge mutagenesis are predicted to reside on the surface of the viral protein (Fig 1A). Following cloning and lentiviral transfer, MRC-5-derived cell lines individually expressing the 24 IE1 mutants in a doxycycline (dox)-inducible fashion (TetR-IE1 cells) were generated.

**Fig 2:**
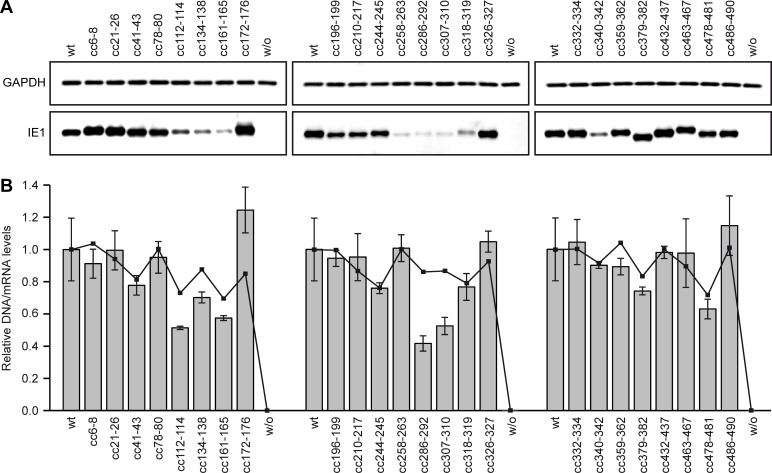
Expression of clustered charge-to-alanine IE1 mutants. (A) TetR cells without (w/o) or with inducible expression of the indicated HA-tagged wild-type (wt) or clustered charge mutant IE1 proteins were treated with dox for 96 h. Whole cell protein extracts were prepared and analyzed by immunoblotting for IE1 (mouse anti-HA) and glyceraldehyde 3-phosphate dehydrogenase (GAPDH). (B) TetR cells without (w/o) or with inducible expression of the indicated HA-tagged wild-type (wt) or clustered charge mutant IE1 proteins were treated with dox for 96 h, and IE1 mRNA levels were determined by RT-qPCR. Results were normalized to TUBB, and means and standard deviations of three experiments are shown in comparison to wt cells (set to 1). Lines represent relative levels of human immunodeficiency virus type 1 (HIV-1) group-specific antigen DNA associated with cellular genomic DNA, determined by qPCR and normalized to cellular ribonuclease P RNA component H1 (RPPH1).

Upon treatment of the TetR-IE1 cell lines with dox, all IE1 proteins mutated in the N- or C-terminal parts outside the core domain accumulated to steady-state levels equal to or higher than the wild-type protein. Likewise, several core domain mutants (IE1cc41-43, cc78-80, cc172-176, cc196-199, cc210-217, cc244-245, cc326-327, cc332-334, cc359-362) produced protein levels comparable to wild-type IE1. However, other core domain mutants (IE1cc112-114, cc134-138, cc161-165, cc258-263, cc286-292, cc307-310, cc318-319, cc340-342) were present at substantially (~50–90%) reduced levels compared to the wild-type protein (Fig 2A). The reduced levels observed for these IE1 mutants likely result from a shorter protein half-life due to core domain misfolding or lack of homo-dimerization. However, some of these mutants also exhibited decreased mRNA levels (Fig 2B, bars), possibly reflecting defective promoter autoregulation, impaired mRNA stability or fewer lentiviral copies (Fig 2B, lines).

The clustered charge-to-alanine scanning indicates that even small mutations in surface residues of the IE1 core domain tend to interfere with protein accumulation to normal levels. Yet, this analysis identifies mutations both in- and outside the core domain that result in stable proteins.

### A subset of IE1 core domain mutants are defective for PML-related activities during interphase and mitosis

**Fig 3:**
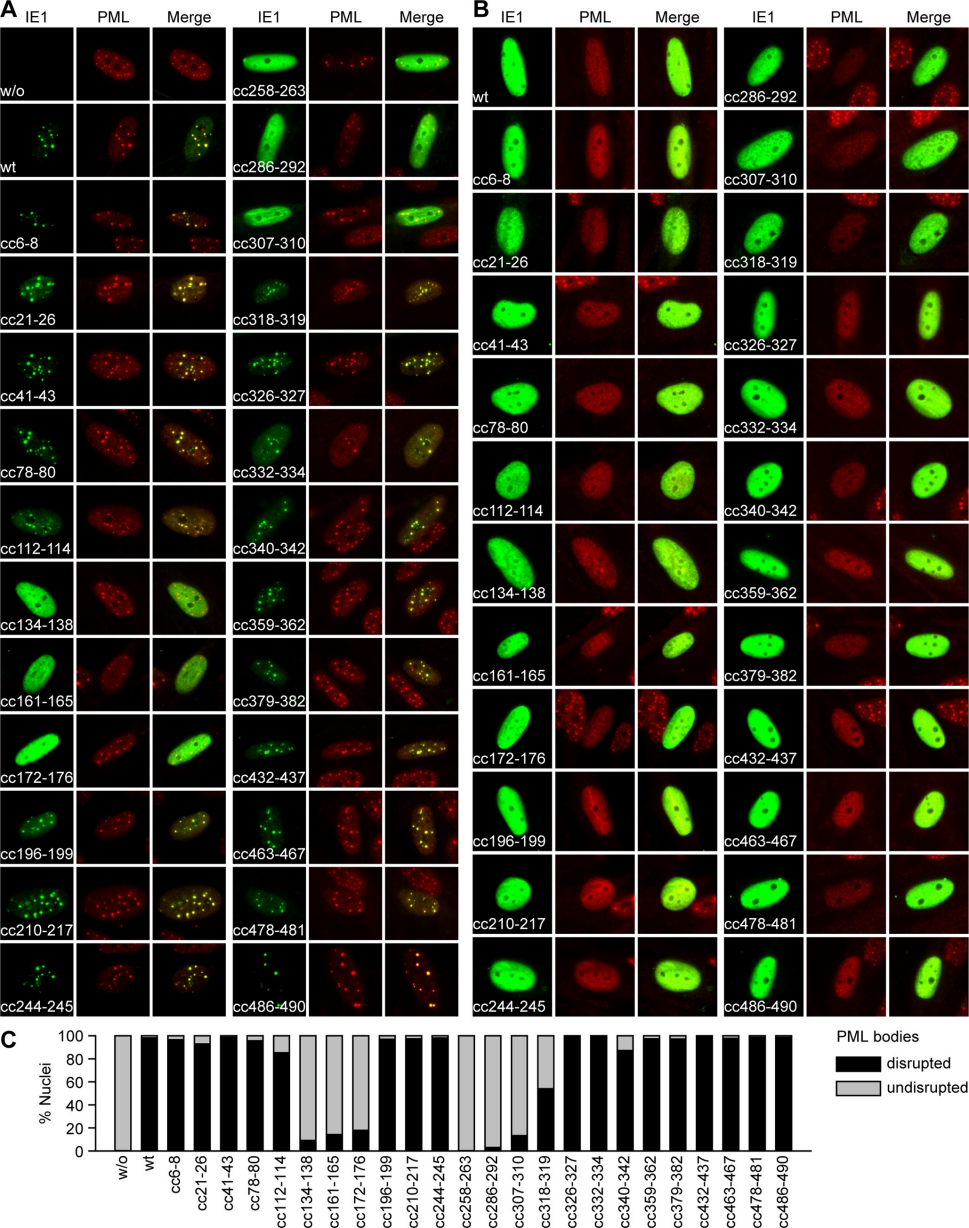
Co-localization with PML and disruption of PML bodies by wild-type and mutant IE1. (A, B) MRC-5 cells were transfected with pCMV.TetO-derived plasmids expressing only the HA tag (w/o) or HA-tagged forms of the indicated wild-type (wt) and clustered charge mutant IE1 proteins. Indirect immunofluorescence staining was performed using mouse anti-HA and rabbit anti-PML combined with goat anti-mouse Alexa Fluor 488 and goat anti-rabbit Alexa Fluor 594 antibodies. Images from interphase cells showing the typical localization of IE1 and PML are presented along with merge images (Leica DMRX microscope, 63× objective). (C) TetR (w/o) and TetR-IE1 cells expressing the indicated wild-type (wt) or clustered charge mutant IE1 proteins were treated with dox for 24 h. Indirect immunofluorescence staining was performed using mouse anti-HA (IE1) and rabbit anti-PML combined with goat anti-mouse Alexa Fluor 488 and goat anti-rabbit Alexa Fluor 594 antibodies. 4′,6-diamidino-2-phenylindole (DAPI) was used to stain DNA. Merge images were taken using a Keyence BZ-9000 microscope (40× objective). Representative images are shown in S1 Fig. The percentage of cells exhibiting predominantly disrupted or intact PML bodies was determined from at least two fields of view (>100 cells) based on manual inspection aided by ImageJ software (National Institutes of Health) [134].

Next, we conducted immunofluorescence microscopy to compare the intracellular localization of wild-type IE1 and the 24 clustered charge mutants. All mutants except for IE1cc21-26 resembled the wild-type protein in localizing predominantly, if not exclusively, to the nucleus (Fig 3A and 3B and S1 Fig). In a subset of cells, the wild-type and 15 of the mutant viral proteins (IE1cc6-8, cc21-26, cc41-43, cc78-80, cc196-199, cc210-217, cc244-245, cc326-327, cc332-334, cc359-362, cc379-382, cc432-437, cc463-467, cc478-481, cc486-490) co-localized with the PML protein in nuclear dots (PML bodies) (Fig 3A). All of these 15 mutants were able to disrupt PML bodies resulting in pan-nuclear staining of both PML and IE1 (Fig 3B and S1 Fig). However, nine mutants carrying substitutions between amino acids 112 and 342 either localized to PML bodies less efficiently than the wild-type protein (IE1cc112-114, cc318-319, cc340-342) or did not at all co-localize with the organelles (IE1cc134-138, cc161-165, cc172-176, cc258-263, cc286-292, cc307-310). Although a subset of cells expressing IE1cc112-114, cc134-138, cc161-165, cc172-176, cc286-292, cc307-310, cc318-319 or cc340-342 exhibited diffuse PML staining, none of these mutants was able to disrupt PML bodies as efficiently as the wild-type protein (Fig 3C and S1 Fig). For example, IE1cc172-176 induced disruption of PML bodies in <20% of cells, while diffuse PML staining was observed in >95% of cells expressing wild-type IE1. IE1cc258-263 failed to disrupt PML bodies in all cells examined.

**Fig 4:**
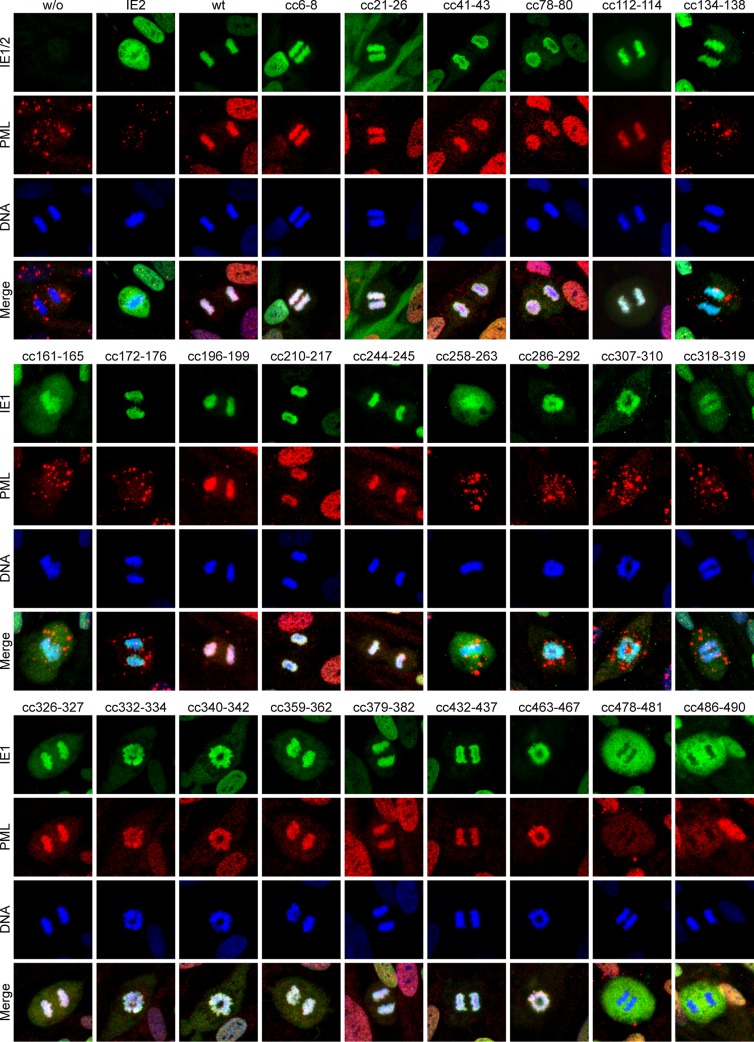
Recruitment of PML to mitotic chromatin and inhibition of MAPP formation by wild-type and mutant IE1. TetR (w/o), TetR-IE2 and TetR-IE1 cells expressing the indicated wild-type (wt) or clustered charge mutant IE1 proteins were treated with dox for 24 h. Indirect immunofluorescence staining was performed using mouse anti-HA and rabbit anti-PML combined with goat anti-mouse Alexa Fluor 488 and goat anti-rabbit Alexa Fluor 594 antibodies. DAPI was used to stain DNA. Images from mitotic cells showing the typical localization of IE1, IE2 and PML relative to DNA are presented along with merge images (Keyence BZ-9000 microscope, 100× objective).

While IE1 exhibits largely pan-nuclear staining in interphase cells, most of the protein is found associated with condensed chromatin in mitotic cells [48, 59, 61, 92, 93]. Immunofluorescence analyses of mitotic cells confirmed that all IE1 mutants except for IE1cc258-263, cc478-481 and cc486-490 exhibited association with mitotic chromatin (Fig 4). IE1cc258-263 appears to be generally inactive for all examined activities, whereas IE1cc478-481 and cc486-490 affect the NBM (amino acids 479–488) and are therefore predicted to be specifically defective for chromatin association [92]. The physical interaction between IE1 and PML is reflected by co-localization of the two proteins at mitotic chromatin [48, 73, 94, 95]. All mutants exhibiting wild-type activity for targeting to and disrupting PML bodies co-localized with PML at mitotic chromatin, except for the two NBM mutants (IE1cc478-481, cc486-490) (Fig 4). Conversely, no co-staining at condensed chromatin was observed between PML and IE1 mutants highly defective in PML body targeting (IE1cc134-136, cc161-165, cc172-176, cc258-263, cc286-292, cc307-310, cc318-319). In the presence of these IE1 mutants, or in the absence of IE1 proteins, PML formed complexes referred to as MAPPs across the mitotic cells (reviewed in [96, 97]). These complexes were also observed in cells expressing IE2, but not when wild-type IE1 was expressed.

These experiments identify residues in the IE1 core domain required for compromising the integrity or preventing the formation of PML complexes during interphase and mitosis by adversely affecting IE1 levels, PML interaction or both.

### IE1cc172-176 encodes a stable protein selectively defective for physical and functional interaction with PML

The immunofluorescence experiments described above identified eight IE1 mutant proteins (IE1cc112-114, cc134-138, cc161-165, cc172-176, cc258-263, cc286-292, cc307-310, cc318-319) that fail to co-localize with PML and are severely defective for PML body disruption as well as inhibition of MAPP formation. However, only IE1cc172-176 produced normal protein levels (Fig 2A) suggesting that the other seven mutations may impose general rather than PML-specific functional defects on IE1. Accordingly, only IE1cc172-176 but neither of the other mutants with PML-related phenotypes were comparable to the wild-type protein in regulating STAT signaling. This conclusion was drawn based on the induction of STAT1- and inhibition of STAT2- or STAT3-stimulated host genes (S2 Fig).

To further characterize the IE1cc172-176 mutant, we compared this protein to previously published IE1 mutants including IE1dl410-420, L174P, L130G/I132G/L133G (also known as YL3) and Y315G/V316G/L317G (also known as YL4). IE1dl410-420 lacks 11 amino acids from the ‘acidic domain’ that encompass the STAT binding motif, and is selectively inactive for regulating IFN- and IL6-type signaling [52]. This deletion was also combined in a double mutant with IE1cc172-176 (IE1cc172-176/dl410-420). The IE1 L174P core domain mutant is broadly defective for all tested functions including PML binding and co-localization, inhibition of PML SUMOylation, PML body disruption and inhibition of PML-mediated transcriptional repression [34, 56, 77, 83]. The core domain mutants IE1 L130G/I132G/L133G and Y315G/V316G/L317G have both been shown to be defective in PML co-localization, PML body disruption, inhibition of PML SUMOylation and functional complementation of infected cell protein 0 (ICP0), a herpes simplex virus 1 protein that shares functional similarities with IE1 [98, 99]. In addition, the core domain mutant IE1dl291-320 was included in the some of the assays. IE1dl291-320 is generally defective in both PML- and STAT-related functions (S3 Fig) [34, 73].

**Fig 5:**
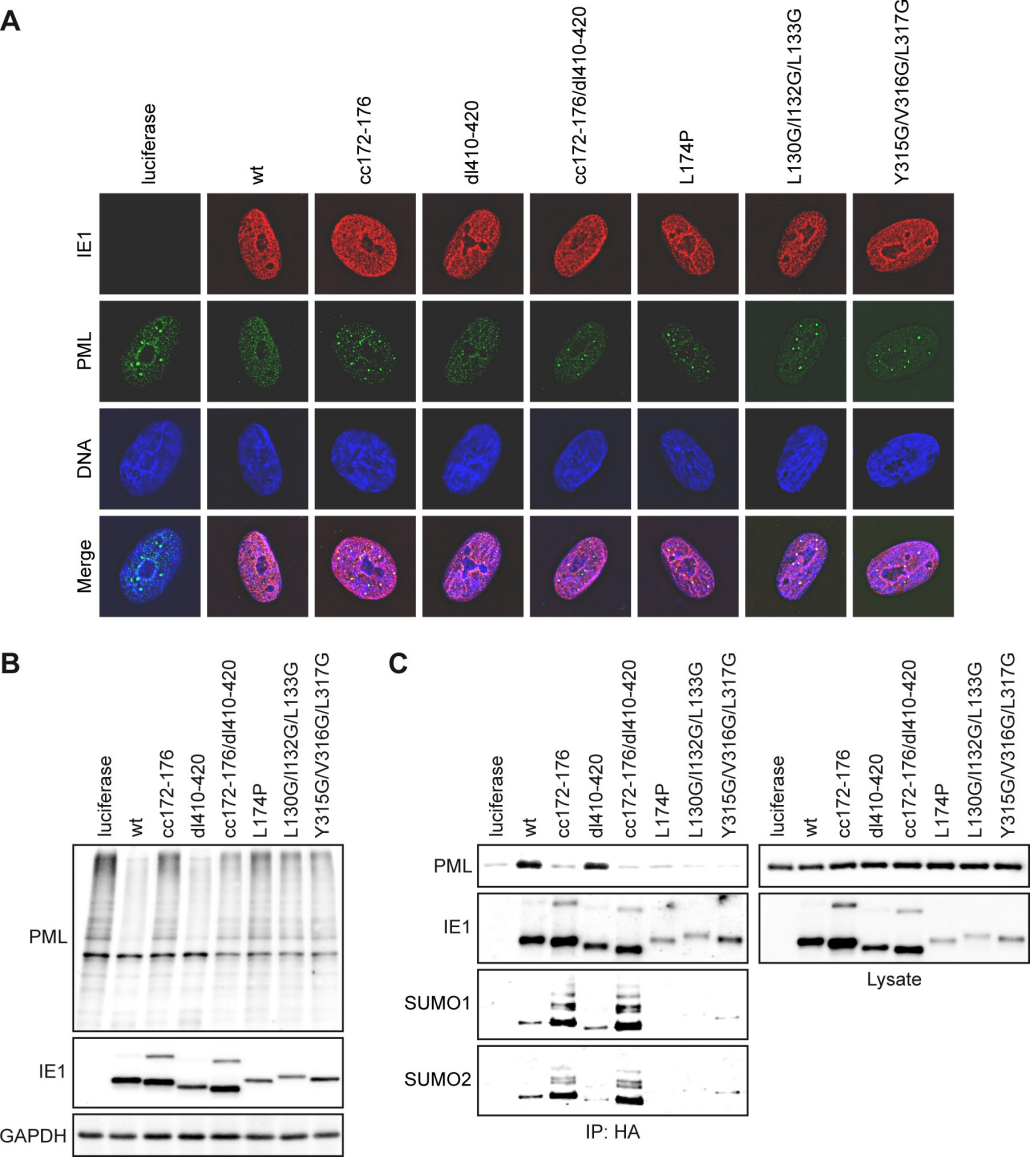
Lack of PML interaction and hyper-SUMOylation of IE1cc172-176. (A) TetOne cells expressing firefly luciferase and TetOne-IE1 cells expressing the indicated HA-tagged wild-type (wt) or mutant IE1 proteins were treated with dox for 24 h. Indirect immunofluorescence staining was performed using mouse anti-HA (IE1) and rabbit anti-PML combined with goat anti-mouse Alexa Fluor 594 and goat anti-rabbit Alexa Fluor 488 antibodies. DAPI was used to stain DNA. Images from interphase cells showing the typical localization of IE1 and PML relative to DNA are presented along with merge images (DeltaVision Restoration Microscope System, 100× objective). (B) TetOne cells expressing firefly luciferase and TetOne-IE1 cells expressing the indicated HA-tagged wild-type (wt) or mutant IE1 proteins were treated with dox for 72 h. Whole cell extracts were prepared in buffer (pH 7.2) with NEM and analyzed by immunoblotting for PML, IE1 (mouse anti-HA) and GAPDH. (C) 293T cells were co-transfected with plasmids encoding PML and firefly luciferase or the indicated HA-tagged wild-type (wt) or mutant IE1 proteins. Cells were fixed with formaldehyde at 48 h post transfection, lysed in buffer (pH 7.2) with NEM and used for immunoprecipitation with anti-HA magnetic beads. Samples of lysates and immunoprecipitates (IP: HA) were analyzed by immunoblotting for PML, IE1 (mouse anti-IE1/1B12), SUMO1 and SUMO2.

Immunofluorescence microscopy confirmed that all tested IE1 mutants localized to the nucleus but only IE1dl410-420 was able to disrupt PML bodies like the wild-type protein (Fig 5A and S3C Fig). We further observed a less efficient reduction in high molecular weight PML species compared to wild-type IE1 in all mutants other than IE1dl410-420 (Fig 5B). Finally, binding to PML above background was exclusively observed for the wild-type and IE1dl410-420 proteins, but for none of the other mutants (Fig 5C). Out of all mutants defective in these assays, only IE1cc172-176 and cc172-176/dl410-420 were present at levels comparable to the wild-type protein, while the levels of IE1 L174P, L130G/I132G/L133G and Y315G/V316G/L317G were substantially lower (Fig 5C). Relative comparison of metabolic stability by temporally controlled IE1 expression further demonstrated that all tested mutants except for IE1cc172-176 exhibit markedly accelerated turnover compared to the wild-type protein (S4 Fig).

To our knowledge, IE1cc172-176 is the first mutant suited to provide specific information about the contribution of PML-related activities to IE1 function and hCMV replication, as it fails to interact with PML but produces a stable protein fully active for other functions. We therefore focused our further analyses on the phenotype of IE1cc172-176.

### IE1 can undergo mixed SUMO chain formation and is SUMOylated at nucleosomes independent of PML binding

**Fig 6:**
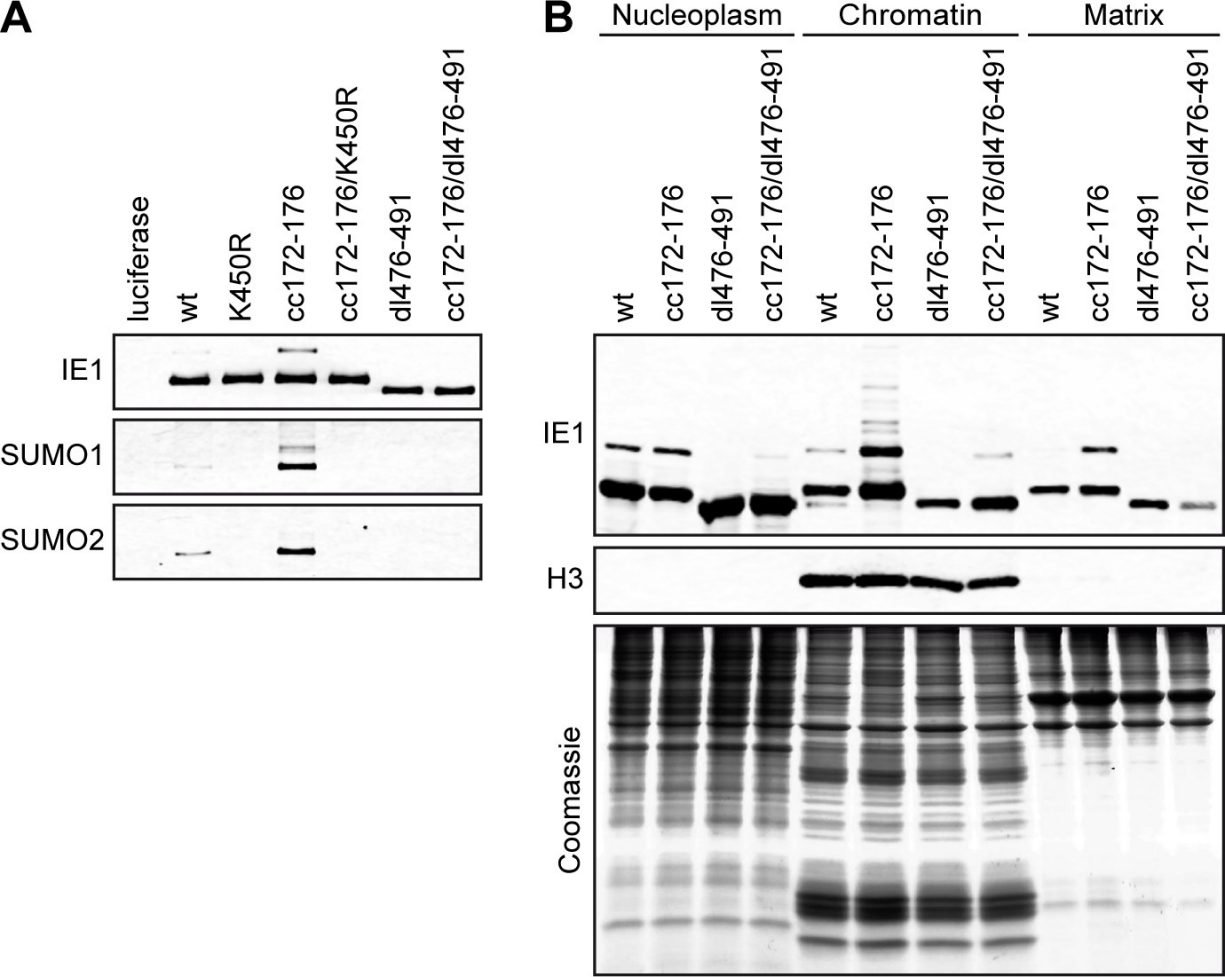
Link between chromatin association and SUMOylation of IE1. (A) TetOne cells expressing firefly luciferase and TetOne-IE1 cells expressing the indicated HA-tagged wild-type (wt) or mutant IE1 proteins were treated with dox for 48 h. Protein extracts prepared in buffer (pH 8.0) with IAA and NEM were used for immunoprecipitation with anti-HA magnetic beads, and samples were analyzed by immunoblotting for IE1 (mouse anti-HA), SUMO1 and SUMO2. (B) TetR-IE1 cells expressing the indicated HA-tagged wild-type (wt) or mutant IE1 proteins were treated with dox for 48 h. Cell nuclei were isolated and fractionated into nucleoplasm, chromatin and matrix in buffers (pH 6.8–7.5) with IAA and NEM. Samples were analyzed by immunoblotting for IE1 (mouse anti-HA) and histone H3, and by Coomassie Brilliant Blue staining for total protein.

It has been established that IE1 can undergo post-translational modification by covalent attachment of a single SUMO1 or SUMO3 moiety to K450 [53–58, 83, 100, 101]. Furthermore, a recent report concluded that PML acts as an E3 ligase for IE1 SUMOylation [58]. Thus, we expected that IE1cc172-176 would exhibit reduced SUMOylation relative to the wild-type protein. However, increased levels of high molecular weight forms most likely corresponding to mono-SUMOylated proteins were detected for IE1cc172-176 and cc172-176/dl410-420 compared to wild-type IE1 (Fig 5B). Further analysis revealed that IE1cc172-176 and cc172-176/dl410-420 are not only hyper-modified by SUMO monomers, but also form polymeric chains that include SUMO1 and SUMO2 (Fig 5C). Hyper-SUMOylation appeared to result specifically from lack of PML binding, since it was not observed in IE1 proteins other than those carrying the cc172-176 mutation (Figs 5B, 5C and 6A). As expected, any detectable SUMO conjugation to IE1 was abolished when K450 was replaced by arginine in either a single mutant (IE1 K450R) or a double mutant (IE1cc172-176/K450R) (Fig 6A). However, unexpectedly, proteins lacking the CTD (IE1dl476-491, cc172-176/dl476-491) required for nucleosome binding turned out to be SUMOylation-deficient as well. Consistent with a link between chromatin targeting and SUMO modification, IE1dl476-491 exhibited reduced localization to chromatin, lack of PML binding (IE1cc172-176) resulted in enhanced chromatin association, and an intermediate phenotype was observed for a double mutant (IE1cc172-176/dl476-491). SUMOylated forms of IE1 were detected across all nuclear compartments including nucleoplasm, chromatin and nuclear matrix (Fig 6B).

These results reveal that SUMOylation of IE1 is nucleosome-based, occurs independent of PML binding and can involve mixed polymeric chains.

### IE1 targets PML and Sp100 via distinct activities

**Fig 7:**
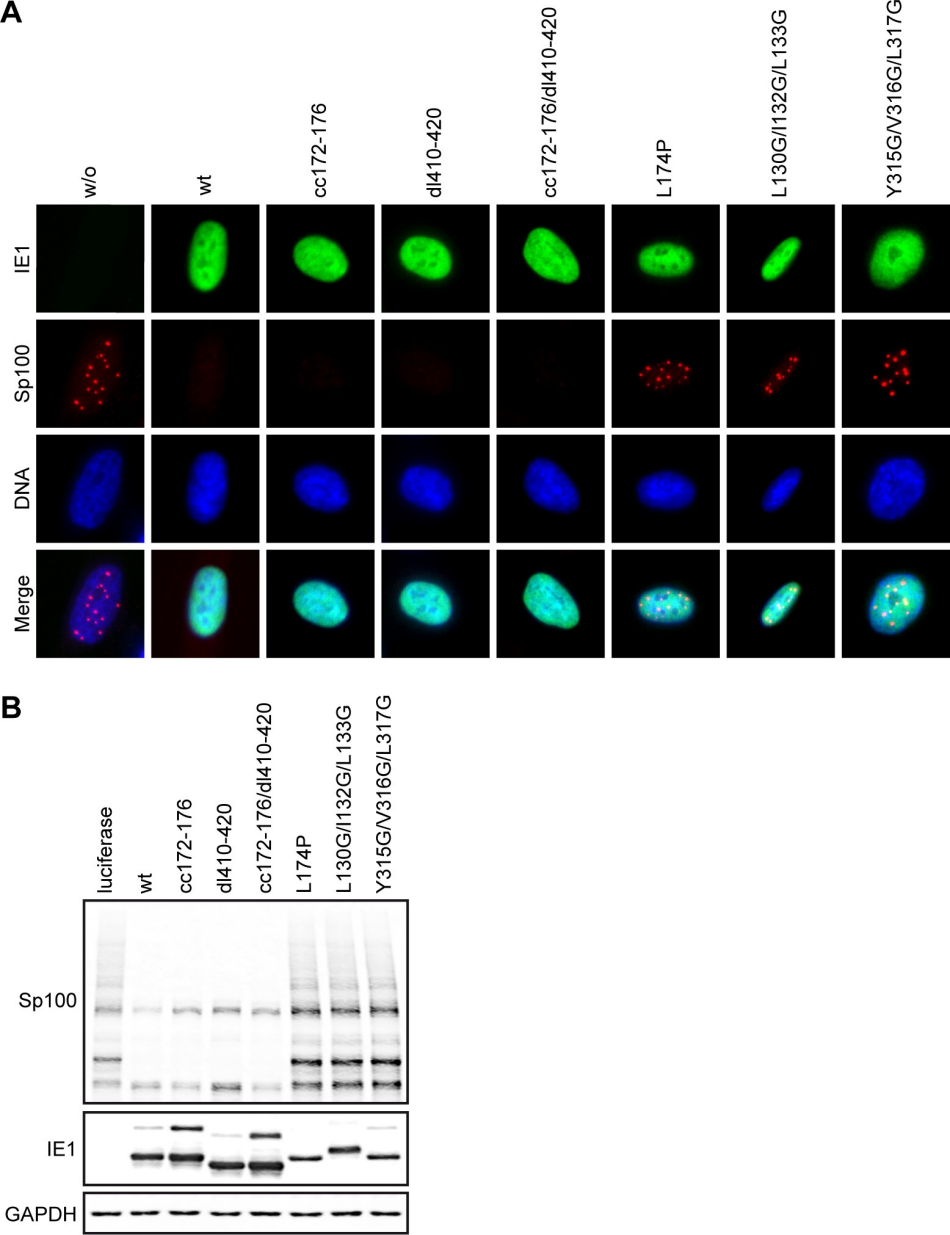
Distinction between Sp100 degradation and PML interaction by IE1. (A) TetR (w/o) and TetR-IE1 cells expressing the indicated HA-tagged wild-type (wt) or mutant IE1 proteins were treated with dox for 24 h. Indirect immunofluorescence staining was performed using mouse anti-HA (IE1) and rabbit anti-Sp100 combined with goat anti-mouse Alexa Fluor 488 and goat anti-rabbit Alexa Fluor 594 antibodies. DAPI was used to stain DNA. Images from interphase cells showing the typical staining pattern of IE1 and Sp100 relative to DNA are presented along with merge images (Keyence BZ-9000 microscope, 100× objective). (B) TetR cells expressing firefly luciferase and TetR-IE1 cells expressing the indicated HA-tagged wild-type (wt) or mutant IE1 proteins were treated with dox for 48 h. Whole cell extracts prepared in buffer (pH 8.0) with NEM were subjected to immunoblotting for Sp100, IE1 (mouse anti-HA) and GAPDH.

Besides PML and SUMO1-3, Sp100A is another constituent of PML bodies targeted by IE1. It has been proposed that IE1 binds to Sp100A, interferes with Sp100A SUMOylation and targets the protein for proteasomal degradation [51, 79, 102]. It is unclear whether the effects IE1 exerts on PML and Sp100A are linked or not. We performed immunofluorescence analyses to monitor how wild-type and mutant IE1 proteins affect the localization and accumulation of Sp100. Sp100 was predominantly identified as nuclear dots both in the absence of IE1 as well as in the presence of mutants that accumulate to low levels and are thought to be broadly defective (IE1 L174P, L130G/I132G/L133G or Y315G/V316G/L317G). In contrast, little Sp100 was detected when wild-type IE1, IE1cc172-176, dl410-420 or cc172-176/dl410-420 were expressed (Fig 7A). Results consistent with these observations were obtained from immunoblotting. Reduced levels of Sp100 were detected in cells expressing wild-type IE1, IE1cc172-176, dl410-420 or cc172-176/dl410-420, but not in those expressing IE1 L174P, L130G/I132G/L133G or Y315G/V316G/L317G. The reduction in Sp100 levels appeared to affect preferentially higher molecular weight (SUMOylated) forms of Sp100, most likely both Sp100A and Sp100B (Fig 7B).

These results indicate that Sp100 targeting by IE1 is a distinct activity unrelated to PML or STAT binding. Thus, IE1 targets the two major structural constituents of PML bodies via physically separable and functionally distinct mechanisms.

### PML targeting by IE1 is dispensable for hCMV replication

**Fig 8:**
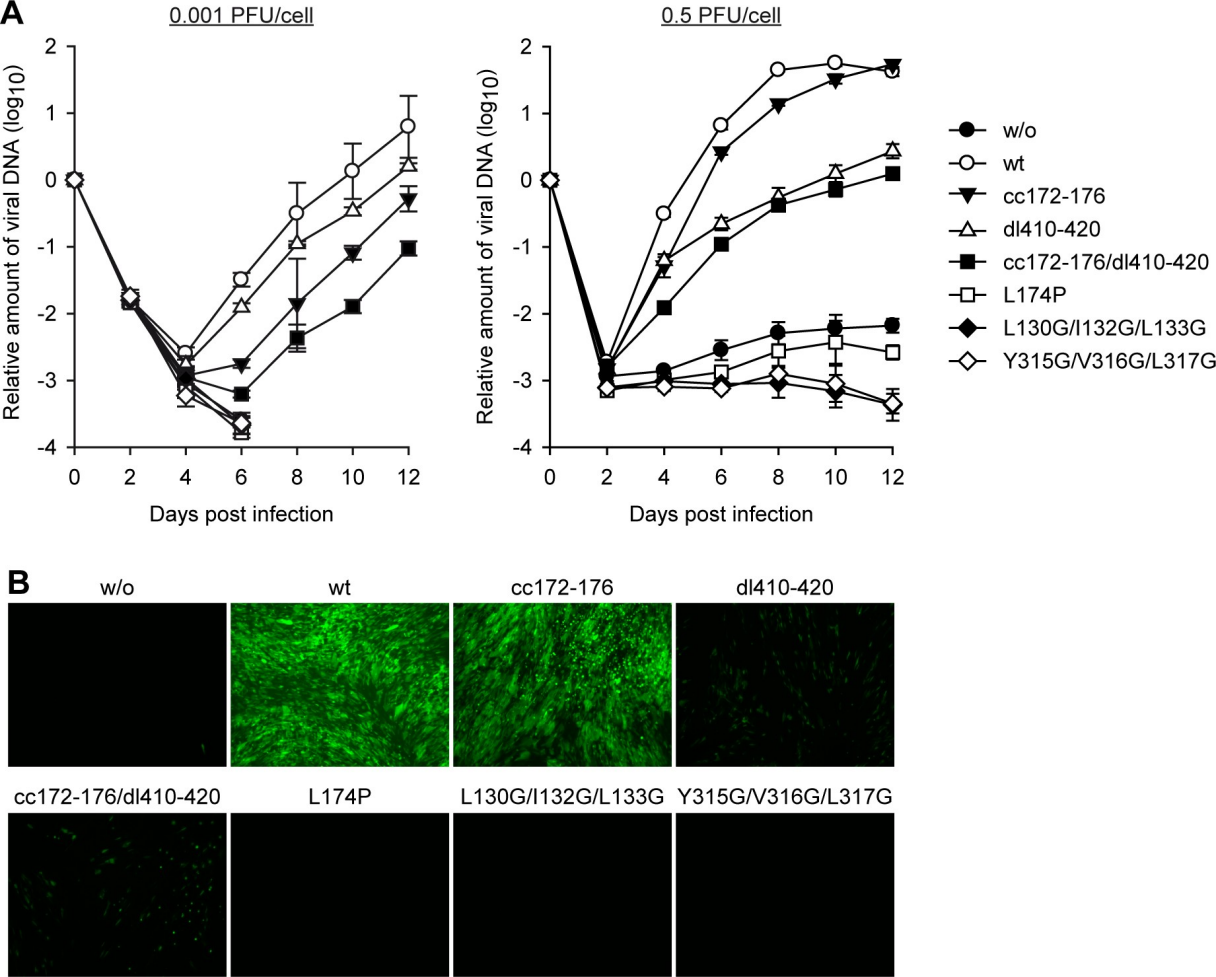
Compensation by IE1cc172-176 for wild-type IE1 in hCMV replication. (A) TetOne cells (w/o) and TetOne-IE1 cells expressing the indicated HA-tagged wild-type (wt) or mutant IE1 proteins were infected with gTBdlIE1 at an MOI of 0.001 PFU/cell (left panel) or 0.5 PFU/cell (right panel). Every 48 h, half of the culture media was replaced and viral replication was assessed by qPCR-based relative quantification of hCMV DNA from culture supernatants with primers specific for UL86. Data are presented as means and standard deviations from three independent infections. (B) TetOne cells (w/o) and TetOne-IE1 cells expressing the indicated HA-tagged wild-type (wt) or mutant IE1 proteins were infected with gTBdlIE1 at an MOI of 0.5 PFU/cell as described in (A), and viral replication was assessed at day 8 post infection by fluorescence microscopy (EVOS FL Cell Imaging System, 4× objective).

The ability to counteract PML has been considered to be crucial for IE1 function and hCMV replication in human fibroblasts, especially at low MOIs. As pointed out above, this perception is largely based on the analysis of metabolically unstable and presumably misfolded mutant proteins including IE1 L174P. To revisit the impact of PML targeting on hCMV replication, TetR-IE1 cell lines expressing wild-type or mutant IE1 and IE1-negative control cells were infected at two different MOIs (0.001 or 0.5 PFU/cell) with IE1-deficient hCMV (gTBdlIE1) expressing the enhanced green fluorescent protein (EGFP). Infection was monitored by quantifying extracellular viral DNA (Fig 8A) and imaging fluorescence emission from EGFP (Fig 8B). As expected, little (MOI = 0.5) or no (MOI = 0.001) replication by gTBdlIE1 was observed in the absence of IE1, but replication was boosted by up to five logs in the presence of wild-type IE1. IE1 L174P, L130G/I132G/L133G or Y315G/V316G/L317G failed to demonstrate any compensatory effect on gTBdlIE1 replication at either MOI consistent with the low protein levels and broad functional defects observed with these mutants. In contrast, attenuated mutant virus replication was rescued to varying degrees by IE1cc172-176, dl410-420 or cc172-176/dl410-420. In fact, gTBdlIE1 replication only modestly differed between cells expressing wild-type IE1 or IE1cc172-176 with roughly one log difference at the lower and almost no difference at the higher MOI. The replication phenotypes linked to lack of PML binding (IE1cc172-176) or STAT binding (IE1dl410-420) appeared to be additive in the double mutant (IE1cc172-176/dl410-420). In contrast to the IE1-PML interaction, the IE1-STAT interaction contributed more significantly to viral replication at the higher rather than the lower MOI. The MOI-dependent phenotype of the IE1dl410-420 mutant may result from increased IFN secretion when a larger number of cells are infected.

These findings indicate that the IE1-PML interaction aids hCMV replication to some extent when cells are infected by single virus particles. However, PML targeting is neither crucial for IE1 function nor for hCMV replication in human fibroblasts, not even at low MOIs.

### PML targeting by IE1 is linked to activation rather than inhibition of antiviral gene expression

Previous work established that IE1 inhibits type I IFN signaling and subsequent induction of ISGs by targeting STAT2 [45, 53, 69]. More recent reports suggested that PML interaction contributes to inhibition of type I IFN signaling and ISG induction by IE1 as well [34, 77]. However, the latter studies relied on IE1 L174P and mutants with large deletions (IE1 1–382, IE1dl291-320) known or expected to produce proteins that are metabolically unstable, globally misfolded, broadly inactive or all of the above (S4 Fig) [34, 49, 50, 77].

**Fig 9:**
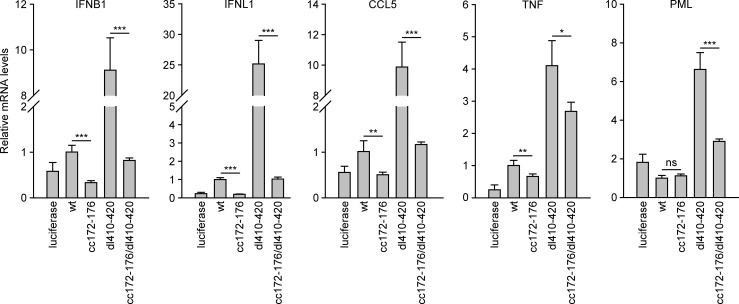
Reduced cytokine and ISG expression during hCMV infection due to lack of IE1-PML interaction. TetOne cells expressing firefly luciferase and TetOne-IE1 cells expressing the indicated HA-tagged wild-type (wt) or mutant IE1 proteins were treated with dox for 48 h and infected with gTBdlIE1 at an MOI of 2 PFUs/cell for 6 h. Relative mRNA levels were determined by RT-qPCR for the indicated cytokine genes (IFNB1, IFNL1, CCL5, TNF) and PML. Results were normalized to TUBB, and means and standard deviations of three biological and two technical replicates are shown in comparison to cells expressing wt IE1 (set to 1). Statistical significance was assessed in Excel using a two-tailed, unpaired T-test; ns, not significant; *, p < 0.05; **, p < 0.01; ***, p < 0.001.

Our initial analysis of IE1cc172-176 in TetR-IE1 cells revealed that this mutant was at least equally efficient as the wild-type protein in inhibiting the induction of OAS1, a prototypical ISG, triggered by IFNα (S1 Fig). To expand on this observation, fibroblasts were induced to express wild-type IE1, IE1cc172-176, dl410-420, cc172-176/dl410-420 or luciferase (control) and infected with gTBdlIE1 for six hours (Fig 9). IE1cc172-176 expression resulted in significantly reduced induction of transcripts from the IFNB1, IFNL1, CCL5 and TNF genes relative to the wild-type protein following infection. Conversely, expression of IE1dl410-420 led to a marked increase in infection-related induction of the IFNB1, IFNL1, CCL5, TNF and PML genes. This increase was greatly diminished when PML and STAT binding was jointly abolished (IE1cc172-176/dl410-420).

**Fig 10:**
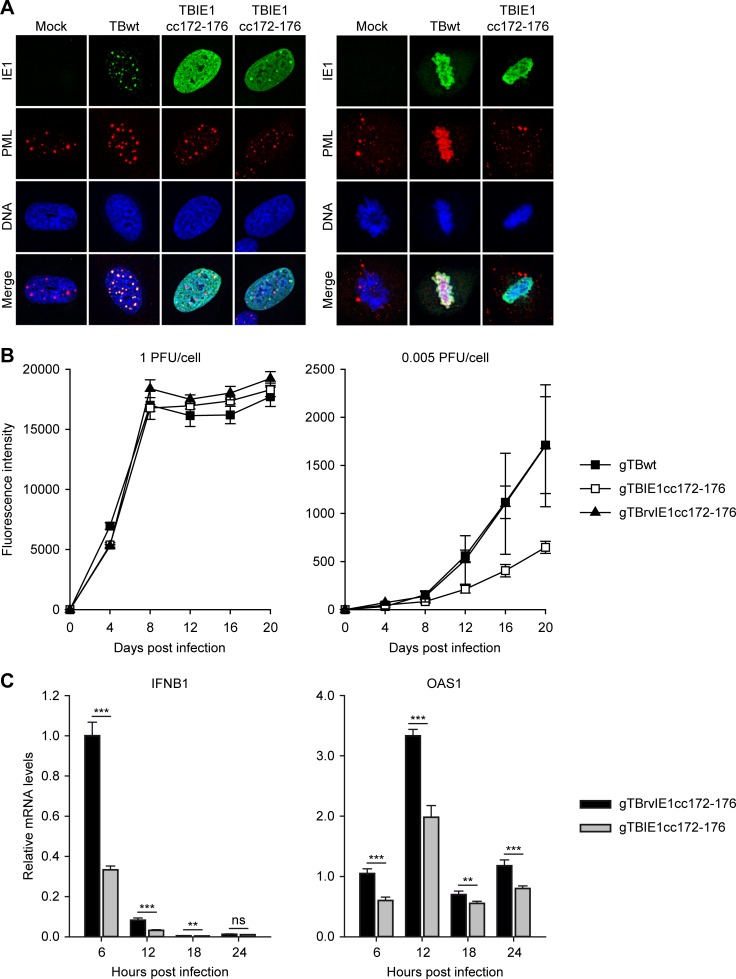
Little attenuation and reduced IFN response in IE1cc172-176 mutant hCMV. (A) MRC-5 cells were mock-infected or infected with hCMV TBwt or TBIE1cc172-176 at an MOI of 0.5 PFU/cell for 24 h. Indirect immunofluorescence staining was performed using mouse anti-IE1 and rabbit anti-PML combined with goat anti-mouse Alexa Fluor 488 and goat anti-rabbit Alexa Fluor 594 antibodies. DAPI was used to stain DNA. Images from interphase (left) or mitotic (right) cells showing the typical localization of IE1 and PML relative to DNA are presented along with merge images (Keyence BZ-9000 microscope, 100× objective). (B) MRC-5 cells were infected with gTBwt, gTBIE1cc172-176 or gTBrvIE1cc172-1176 at an MOI of 1 PFU/cell (left) or 0.005 PFU/cell (right). Virus replication was monitored every 4 days by measuring fluorescence intensity in a Tecan Infinite M200 PRO microplate reader. Mean values and standard deviations of three (gTBwt, gTBrvIE1cc172-176) or six (gTBIE1cc172-176) infections are shown. (C) MRC-5 cells were infected with gTBrvIE1cc172-176 or gTBIE1cc172-176 at an MOI of 2 PFUs/cell. At the indicated times post infection, relative mRNA levels were determined by RT-qPCR for the IFNB1 and OAS1 genes. Results were normalized to TUBB, and means and standard deviations of three biological and two technical replicates are shown in comparison to cells infected with gTBrvIE1cc172-176 for 6 h (set to 1). Statistical significance was assessed in Excel using a two-tailed, unpaired T-test; ns, not significant; **, p < 0.01; ***, p < 0.001.

To investigate these findings further, mutant hCMV strains expressing IE1cc172-176 (TBIE1cc172-176 and gTBIE1cc172-176) were generated and compared with corresponding wild-type and revertant viruses. As expected, IE1cc172-176 expressed from the mutant virus did not co-localize with PML, neither at PML bodies nor at mitotic chromatin (Fig 10A), and both TBIE1cc172-176 and gTBIE1cc172-176 were severely defective for PML body disruption (S5 Fig). Consistent with the results from our trans-complementation assays, the replication kinetics of gTBIE1cc172-176 were slightly delayed at low MOI (0.005 or 0.5 PFU/cell) compared to the wild-type and revertant viruses, but only by a factor of less than three (Fig 10B and S6 Fig). At a higher MOI (1 PFU/cell), the mutation had no significant effect on viral replication (Fig 10B). We subsequently compared the kinetics of transcript accumulation related to IFNB1, the major type I IFN expressed in fibroblasts, and OAS1 in cells infected with gTBIE1cc172-176 or a revertant virus for six to 24 hours. Again, IFNB1 and OAS1 mRNA levels were lower in the mutant compared to the revertant virus infection at several time points (Fig 10C).

These results confirm that the interaction between IE1 and PML is dispensable for hCMV replication and demonstrate that efficient inhibition of ISG expression by IE1 does not depend on this interaction. Instead, the findings suggest that the IE1-PML interaction is linked to enhanced rather than reduced expression of cytokines and ISGs. These observations are consistent with the idea that the disruption of PML bodies by IE1 contributes to the antiviral and proinflammatory response during hCMV infection.

## Discussion

PML bodies have fascinated cell biologists and molecular virologists due to their beauty and apparent involvement in many cellular processes including viral infection. Major evidence for a role of PML bodies and their proteins in the cellular defense against viruses has come from the identification and study of viral antagonists. The hCMV IE1 protein is one of the most prominent antagonists of PML bodies and interacts with several constituents of these organelles including isoforms of SUMO, Sp100 and PML. Binding to PML and disruption of PML bodies have been considered pivotal to IE1 function and hCMV replication, at least upon low MOI infection. However, this perception has relied largely on the analysis of mutant proteins and viruses affecting the IE1 core domain. Given the disruptive and destabilizing nature of many published IE1 mutants and the lack of mutants specifically targeting PML interaction, we conducted systematic clustered charge-to-alanine scanning along the viral protein. To our knowledge, this is a novel approach that has not been pursued before to explore IE1 function. Phenotypic screening of the mutant library resulted in the identification of IE1cc172-176, a stable protein selectively inactive for physical and functional interaction with PML. Further investigation of IE1cc172-176 led to several unexpected new findings relevant to PML function, IE1 activity and hCMV replication with general implications for mechanisms of antiviral defense including innate immune activation.

Deletions in the putatively disordered N-terminal domain (amino acids 1–24), encoded by exon 2 of the major immediate gene, did not adversely affect IE1 protein accumulation in a previous study [46]. Likewise, the clustered charge mutants IE1cc6-8 and IE1cc21-26 produced slightly higher rather than lower protein levels (Fig 2A). Although the N-terminal domain contains a nuclear localization signal (NLS) [46, 48], all IE1 mutants resembled the wild-type protein in localizing to the nucleus (Fig 3A and 3B). However, nuclear accumulation of IE1cc21-26 but not IE1cc6-8 was less efficient compared to wild-type IE1. Three clustered charge mutations (IE1cc326-327, cc332-334, cc340-342) coincide with a second NLS reported between amino acids 326 and 342 in IE1 [47]. Of the three mutants, only IE1cc332-334 showed slightly less efficient nuclear localization compared to the wild-type protein. These observations suggest that basic residues K21, R24 and K332 may be critical for nuclear import of IE1. Moreover, the NBM we previously defined [103] may represent a nuclear retention signal adding to what appear to be multiple mechanisms of IE1 nuclear localization. Many functional analyses of the IE1 core domain (amino acids 25–378), encoded by exons 3 and 4 of the major immediate-early gene, have relied on mutants with relatively large deletions including IE1dl291-320 [46, 48, 56, 73, 103–105]. Other studies of this domain have involved the single but disruptive amino acid substitution L174P [56, 77, 83, 84, 105]. In general, these mutant proteins proved to be metabolically unstable and broadly non-functional, most likely due to global misfolding. For example, the L174P mutation disrupts the structural integrity of IE1 and reduces the estimated protein half-life from >30 to only 5 hours [77]. Accordingly, IE1 L174P exhibited reduced protein accumulation, accelerated turnover and broad loss of function in our study (Figs 5, 7 and 8 and S4 Fig). Even more subtle mutations in the IE1 core domain, such as the L130G/I132G/L133G and Y315G/V316G/L317G substitutions described earlier [98, 99] and examined in this study, produced proteins that behave similar to IE1 L174P in terms of accumulation, stability and function (Figs 5, 7 and 8 and S4 Fig). Likewise, most of our own mutations in the IE1 core domain were linked to diminished protein levels compared to the wild-type, although clustered charge-to-alanine substitutions are not expected to disrupt overall protein structure [85–90]. With one notable exception (IE1cc172-176), all clustered charge mutations introduced between residues 112 and 319 reduced IE1 protein levels to some extent. IE1cc112-114 and IE1cc134-138 affecting central or distal parts of predicted Helix3, respectively, accumulated to substantially lower levels (Figs 1 and 2A). This finding is in line with previous reports showing that deletion of amino acids 1 to 85 or substitution of residues between positions 130 and 133 (IE1 L130G/I132G/L133G), both within predicted Helix3, lead to diminished protein accumulation [46, 98, 99]. Likewise, clustered charge mutations affecting predicted Helix5 (IE1cc161-165) as well as predicted Helix8 and Helix9 between residues 258 and 319, including Y315G/V316G/L317G, came with profoundly reduced protein levels (Figs 1 and 2A). Our previous mutational analyses of IE1 have focused on the presumably disordered ‘acidic domain’ (amino acids 379–475), including the SBM and SUMOylation motif, and on the CTD/NBM (amino acids 476–491) [45, 51, 52, 55, 92]. Small mutations in this C-terminal quarter of IE1 generally produce stable proteins. Consequently, normal protein levels were observed for all clustered charge IE1 mutants between amino acids 379 and 490 (Fig 2A). In fact, none of the substitutions downstream of residue 325 adversely affected protein accumulation except for IE1cc340-342, suggesting that mutations in predicted Helix11 but not Helix10 may be tolerated (Figs 1 and 2A).

**Fig 11:**
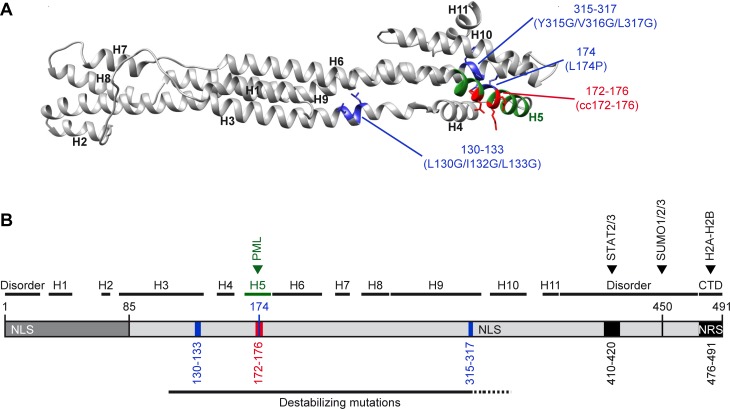
Helix5 as determinant of PML interaction and structure-stability relationship of IE1. (A) Tertiary protein structure of hCMV IE1 core domain modelled on the orthologous domain of rhCMV IE1 (PDB 4WID) [49] using Phyre2. The predicted α-helices (H1-H11) are indicated, including H5 involved in PML interaction shown in green. The residues including side chains relevant to mutations examined in this study are shown in blue and red. (B) Schematic of hCMV IE1 showing structural organization, including N-terminal segment shared with IE2 (amino acids 1–85), putative disordered regions, predicted α-helices (H1-H11) including H5 involved in PML interaction (green) and CTD. Residues relevant to mutations examined in this study (blue and red), binding sites for cellular proteins (PML, STAT2/STAT3, SUMO1-3, H2A-H2B) as well as two NLS and a putative nuclear retention signal (NRS) are shown alongside these structural features and select amino acid positions. The segment of the IE1 core domain (H3-H9/H10) proposed to be particularly sensitive to protein destabilizing mutations is indicated as well.

The low protein levels observed for many of the IE1 core domain mutants may partly relate to defects in promoter autoregulation or mRNA stability (Fig 2B). Some of the mutations, especially those in Helix8 and Helix9, might interfere with homo-dimerization potentially resulting in shortened protein half-lives in case IE1 dimers are more stable than monomers [50]. However, many mutations likely cause disruption to the overall fold as illustrated by the three-dimensional structure of the hCMV IE1 core modelled on the orthologous domain of rhCMV IE1 [49, 106]. The 11 α-helices and other elements of the IE1 core domain are arranged in coiled-coil bundles forming one single structural unit. Consequently, many mutations targeting the core domain will generate misalignment of secondary structure elements disrupting the fold. For example, L174 is located in the hydrophobic core within predicted Helix5. This residue stabilizes the fold between Helix5, Helix9 and Helix10. Mutation to proline is predicted to disrupt the stabilizing interactions and to induce helix redirection explaining the broad functional defects linked to IE1 L174P. A similar situation applies to IE1 L130G/I132G/L133G and Y315G/V316G/L317G. In contrast, the charged residues (amino acids 172–173 and 175–176) replaced in IE1cc172-176 are expected to be on the protein surface and unlikely to participate in interactions between helices (Fig 11). Consequently, IE1cc172-176 produces a metabolically stable and broadly active protein (Figs 2–10, S1 Fig and S4 Fig).

PML-related activities are generally preserved in most C-terminal and some N-terminal IE1 mutants, but not in mutants involving the core domain [48, 73, 77, 103]. Although the IE1-PML interaction has been proposed to involve an extended interface across the core domain [49, 106], our study identifies predicted Helix5 comprising 18 residues (amino acids 164–181, REMWMACIKELHDVSKGA) to be a critical structural element in the IE1-PML interaction. To our knowledge, IE1cc172-176 is the only existing mutant that specifically probes the interaction with PML. This mutant accumulates to normal steady-state levels (Fig 2A), localizes to the nucleus (Figs 3A, 3B, 5, 6B, 7A and 10A and S2 Fig), associates with chromatin (Figs 4, 6B and 10A), undergoes SUMOylation (Figs 5B, 5C, 6 and 7B) and retains the ability to regulate STAT1-, STAT2- and STAT3-dependent gene expression differentially (S1 Fig). However, this mutant does not co-localize with PML, neither at PML bodies during interphase nor at MAPPs and chromatin during mitosis (Figs 3A, 4 and 10A). Moreover, IE1cc172-176 neither specifically binds to PML nor significantly inhibits PML SUMOylation (Fig 5B and 5C). These observations strongly suggest that IE1cc172-176 lost any affinity for PML and consequently lacks all PML-related functions. This conclusion seems to clash with the fact that IE1cc172-176 is largely but not completely inactive for PML body disruption (Fig 3 and S2 Fig). Rather than implying residual PML binding by IE1cc172-176 below the detection limit of our assays, we propose that IE1 employs at least two mechanisms for disrupting PML bodies. The major mechanism depends on PML binding while some PML body disruption by IE1 does not seem to require this interaction. Whether degradation of Sp100 or more indirect effects linked to IE1 expression result in the disruption of PML bodies in a subset of cells remains to be determined.

Given the key role ascribed to PML in antiviral intrinsic immunity, it comes as a surprise that replication of an IE1cc172-176 mutant is only slightly attenuated relative to the wild-type virus (Figs 8 and 10B and S6 Fig). While the lack of mutant virus attenuation at MOIs ≥1 may be due to saturation of the PML-based intrinsic repression system [9, 107], the minor replication defects at MOIs ≤0.5 are less readily explained. We propose that this finding reflects redundant mechanisms employed by hCMV in dealing with PML bodies and their restriction factors. In fact, a number of hCMV proteins other than IE1 including IE2, LUNA, pUL3, pUL35, pUL80a, pUL82 (pp71), pUL83 (pp65), pUL97 and pUS32 have been shown to target PML bodies [75, 108–117]. Having said that, our findings suggest that interactions with STAT family members via the SBM contribute more significantly to IE1 function during productive hCMV infection than PML interaction, especially at higher MOIs. This conclusion aligns with the idea that the IE1-STAT2 interaction promotes hCMV replication by conferring resistance to IFNα and IFNβ [45, 53, 69].

Besides mediating intrinsic immunity, PML has been implicated in activating innate immunity (reviewed in [29, 30]). Certain nuclear PML isoforms are positive regulators of IFN synthesis, and PML may directly promote induction of some ISGs [29, 31–34]. Moreover, it has been proposed that IE1 inhibits ISG activation in part by interacting with PML [34, 77]. We therefore expected that IE1cc172-176 would be deficient in inhibiting ISG expression compared to wild-type IE1. However, we observed lower instead of higher levels of IFN, TNF and ISG expression with the mutant relative to the wild-type protein upon hCMV infection (Figs 9 and 10). This finding is consistent with the idea that disruption of PML bodies is linked to activation rather than inhibition of antiviral gene expression. Activation of antiviral genes following disruption of PML bodies might be mediated by PML isoforms redistributed from the organelles into the nucleoplasm. Alternatively, viral genomes released from repression imposed by PML bodies and physically liberated from these organelles might trigger the activation of nuclear DNA sensors such as IFN-inducible protein 16 (IFI16) or cyclic GMP-AMP synthase (cGAS) to induce an antiviral response. However, these are merely speculations at this point that need to be experimentally tested. Nonetheless, we propose that disruption of PML bodies may link relief from intrinsic immunity to induction of innate immunity.

IE1 has been identified in physical complexes with not only PML [73] but with at least four other constituent proteins of PML bodies including Daxx [80], Sp100A [79], SUMO1 [56] and SUMO3 [58]. Our results demonstrate that IE1 targets Sp100 irrespective of PML binding (Fig 7). Thus, proteasomal degradation of Sp100 is unlikely a consequence of PML body disruption but rather presents a distinct IE1 activity that deserves further attention. Several groups have reported that a proportion of IE1 is modified at residue K450 by single moieties of SUMO1 or SUMO3, two of five known SUMO paralogs [53–58, 83, 94, 100]. We added SUMO2 to this list (Figs 5C and 6A). SUMO2 and SUMO3 share around 95% sequence identity and are often referred to jointly as SUMO2/3. SUMO2/3 forms poly-SUMO chains since residue K11 confers conjugation to itself or other SUMO paralogs. SUMO1, which exhibits only around 50% sequence identity with SUMO2/3, lacks K11 and is therefore conjugated only as a monomer or as a terminator at the end of a poly-SUMO chain [16, 118]. Based on our immunoblotting results (Figs 5C and 6A), we propose that mixed polymeric chains of SUMO2/3 terminated by SUMO1 form at residue K450 of IE1cc172-176. Previous work has shown that SUMOylation of IE1 interferes with binding to STAT2 [53]. It is tempting to speculate about differential effects mono- and poly-SUMOylation may have on IE1 protein interactions beyond STAT2. Although we cannot rule out that poly-SUMOylation is a feature specific to IE1cc172-176, the chains likely exist on the wild-type protein as well but exceed the limit of detection only in the hyper-SUMOylated mutant. Hyper-SUMOylation may result from a difference in subnuclear localization between IE1cc172-176 and the wild-type protein. IE1 usually localizes across several nuclear compartments including nucleoplasm, matrix and chromatin (Fig 6B). A dynamic equilibrium likely exists between the nuclear locations of IE1, which may in part be determined by IE1-PML complex formation in the nucleoplasm and nucleosome binding at chromatin. In this scenario, chromatin association by IE1 would increase on disruption of PML binding. This prediction is consistent with the results from our subnuclear fractionation analysis demonstrating decreased nucleoplasmic and increased chromatin-associated localization of IE1cc172-176 compared to wild-type IE1 (Fig 6B). In turn, increased chromatin association correlates with enhanced SUMOylation and detection of SUMO chains on IE1cc172-176. Conversely, IE1 exhibiting lack of nucleosome binding (IE1dl476-491) and decreased chromatin association is not detectably SUMO-modified (Figs 5C and 6). These observations indicate that SUMOylation of IE1 occurs mostly if not exclusively at nucleosomes. This conclusion is consistent with previous findings suggesting that PML bodies are not the place of IE1 SUMOylation [58] and imply a nucleosome-based E3 SUMO ligase for the viral protein. A previous study concluded that PML serves as an E3 ligase for IE1 SUMOylation [58], and IE1 recruits PML to chromatin (Figs 4 and 10A) [94, 95]. However, our findings challenge the idea that PML is a major SUMO E3 ligase for IE1, since IE1cc172-176 exhibits increased rather than decreased SUMOylation (Figs 5B, 5C, 6 and 7B). Thus, PIAS1 is a more likely SUMO E3 ligase for IE1. PIAS1 has been shown to interact with IE1 and to enhance IE1 SUMOylation [57]. PIAS1 serves as a SUMO E3 ligase for numerous transcription factors and other chromatin-associated proteins including high mobility group nucleosomal binding domain 2 (HMGN2) [119]. SUMOylation by PIAS1 reduces the binding affinity of HMGN2 to nucleosome core particles [119]. Given that both HMGN2 and IE1 target histones H2A-H2B on the nucleosome surface, we are tempted to speculate that PIAS1-mediated SUMOylation might interfere with chromatin association by the viral protein. In fact, our observation that SUMOylated IE1 forms at nucleosomes but localizes throughout the nucleus (Fig 6B) implies a mechanism releasing the viral protein from chromatin.

The findings presented in this study challenge predominant views about IE1, a pivotal viral protein with a crucial function in hCMV replication and pathogenesis. Several of our conclusions should also be more broadly relevant to cellular events beyond hCMV infection given the versatile roles of PML and PML bodies in health and disease (reviewed in [120, 121]).

## Materials and methods

### Plasmids, mutagenesis and cloning

The eukaryotic expression and subcloning vector pCMV.TetO.HA-IE1 encodes wild-type hCMV (Towne) IE1 linked to an N-terminal hemagglutinin (HA) epitope tag expressed from a modified hCMV major immediate-early enhancer-promoter containing tetracycline operator (TetO) sequences [52]. Clustered charge-to-alanine scanning mutagenesis of the IE1 sequence in pCMV.TetO.HA-IE1 was performed using the QuikChange strategy with primers listed in Table 1. QuikChange site-directed mutagenesis was also used to generate plasmids pCMV.TetO.HA-IE1L174P, pCMV.TetO.HA-IE1L130G/I132G/L133G, pCMV.TetO.HA-IE1Y315G/V316G/L317G and pCMV.TetO.HA-IE1K450R from template pCMV.TetO.CMV.HA-IE1 as well as plasmid pCMV.TetO.HA-IE1cc172-176/K450R from template pCMV.TetO.HA-IE1cc172-176. The negative control vector pCMV.TetO.HA-2×Stop-IE1 was constructed by replacing the first two triplets of the IE1 coding sequence with stop codons as described [52]. To generate pCMV.TetO.HA-IE2 encoding the hCMV 86-kDa IE2 protein linked to an N-terminal HA tag, the IE2 cDNA was PCR-amplified from plasmid pCGN-IE2 [122] with oligonucleotide primers #809 and #918 (Table 1) and ligated to *Hin*dIII- and *Eco*RI-digested pCMV.TetO.cIE1 [72]. For each new pCMV.TetO.HA-IE1 or pCMV.TetO.HA-IE2 construct, the entire insert sequence was verified by Sanger sequencing using primers listed in Table 1. The lentiviral plasmid pLKOneo.CMV.EGFPnlsTetR encoding the tetracycline repressor (TetR) linked to a nuclear localization signal (nls) and EGFP [123] was kindly provided by Roger Everett (University of Glasgow). The lentiviral vector pLKO.DCMV.TetO.HA-IE1 expressing HA-tagged wild-type IE1 under positive control of the EGFPnlsTetR protein negatively regulated by dox has been described, as has been plasmid pLKO.DCMV.TetO.HA-IE1dl410-420 encoding a STAT-binding deficient HA-IE1 deletion mutant [52]. Variants of these plasmids encoding HA-tagged IE1 point mutants or HA-IE2 were constructed by replacing the fragment released by *Nde*I and *Eco*RI digestion from plasmid pLKO.DCMV.TetO.cIE1 [72] with the corresponding DCMV-HA-IE1/2 sequence of the pCMV.TetO subcloning vectors described above. Plasmid pLKO.DCMV.TetO.HA-IE1cc172-176/dl410-420 encoding an HA-tagged IE1 double mutant deficient for both PML and STAT binding was generated by inserting the *Bcl*I fragment of pLKO.DCMV.TetO.HA-IE1dl410-420 into plasmid pLKO.DCMV.TetO.HA-IE1cc172-176. The single-plasmid, lentiviral vector pLVX-TetOne-Puro which encodes the dox-activated Tet-On 3G transactivator protein and places the gene of interest under the tight control of a TRE3G promoter was obtained from Clontech alongside control vector pLVX-TetOne-Puro-Luc for dox-inducible expression of firefly luciferase. Recombinant pLVX-TetOne-Puro plasmids encoding HA-tagged IE1 proteins were generated by PCR amplification of insert DNA from pLKO.DCMV.TetO-HA-IE1 plasmids with oligonucleotide primers #1175 and #1176 and inserted into the *EcoR*I- and *Bam*HI-digested parental vector pLVX-TetOne-Puro. Plasmids pLVX-TetOne-Puro-HA-IE1 and pLVX-TetOne-Puro-HA-IE1dl410-420 have been described [124]. To generate pLVX-TetOne-Puro constructs encoding C-terminally truncated IE1 proteins lacking the CTD (HA-IE1dl476-491 and HA-IE1cc172-176/dl476-491) oligonucleotide #1177 was used as reverse primer for PCR amplification. For pLVX-TetOne-Puro-HA-IE1dl291-320, the sequence encoding IE1dl291-320 was PCR-amplified from template pSG5-HA-IE1(dl291-320) [105], kindly provided by Jin-Hyun Ahn (Sungkyunkwan University), using primers #483 and 694, and inserted into pCMV.TetO.cIE1 via *Hin*dIII and *Eco*RI. The IE1dl291-320 sequence was PCR-amplified from the resulting plasmid using primers #1175 and 1176, and ligated to *Bam*HI- and *Eco*RI-digested pLVX-TetOne-Puro. For each new construct, the entire IE1-specific sequence was verified by Sanger sequencing. The pMD2.G and psPAX2 packaging vectors for lentivirus production were obtained from Addgene (plasmids #12259 and #12260, respectively).

**Table 1:** Oligonucleotides used in this study.

#	Sequence (5‘→3‘)	Use
483	TTGCAAAGCTTATGGAGTCCTCTGCCAAGAGAAAG	PCR cloning IE1dl291-320
694	GATACTGAATTCTTACTGGTCAGCCTTGCTTCTAGT	PCR cloning IE1dl291-320
810	CTGACTACGCCGAGTCCTCTGCCGCGGCAGCGATGGACCCTGATAATCCTGACG	QuikChange IE1cc6-8
811	CGTCAGGATTATCAGGGTCCATCGCTGCCGCGGCAGAGGACTCGGCGTAGTCAG	QuikChange IE1cc6-8
840	GCCCTTCCTCCGCGGTGCCAGCGCCCGCGACACCCGTGA	QuikChange IE1cc21-26
841	TCACGGGTGTCGCGGGCGCTGGCACCGCGGAGGAAGGGC	QuikChange IE1cc21-26
812	GACGTTCCTGCAGACTATGTTGGCGGCGGCGGTTAACAGTCAGCTGAGTCTG	QuikChange IE1cc41-43
813	CAGACTCAGCTGACTGTTAACCGCCGCCGCCAACATAGTCTGCAGGAACGTC	QuikChange IE1cc41-43
814	GATTGCAACGAGAACCCCGCGGCAGCTGTCCTGGCAGAACTCGTC	QuikChange IE1cc78-80
815	GACGAGTTCTGCCAGGACAGCTGCCGCGGGGTTCTCGTTGCAATC	QuikChange IE1cc78-80
816	ATGCTGAAAAAATATACCCAGACGGCAGCGGCATTCACTGGCGCCTTTAATATGATG	QuikChange IE1cc112-114
817	CATCATATTAAAGGCGCCAGTGAATGCCGCTGCCGTCTGGGTATATTTTTTCAGCAT	QuikChange IE1cc112-114
842	ATGTTTGCAGAATGCCTTAGATATCTTAGCTGCGGTTGCTGCGCCTTTCGAGGAGATGAAGTGTATTGG	QuikChange IE1cc134-138
843	CCAATACACTTCATCTCCTCGAAAGGCGCAGCAACCGCAGCTAAGATATCTAAGGCATTCTGCAAACAT	QuikChange IE1cc134-138
818	GCATGTATGAGAACTACATTGTACCTGCGGCTGCGGCGGCGATGTGGATGGCTTGTATTAAGGAGCT	QuikChange IE1cc161-165
819	AGCTCCTTAATACAAGCCATCCACATCGCCGCCGCAGCCGCAGGTACAATGTAGTTCTCATACATGC	QuikChange IE1cc161-165
844	GCGGGAGATGTGGATGGCTTGTATTGCGGCGCTGGCTGCTGTGAGCAAGGGC	QuikChange IE1cc172-176
845	GCCCTTGCTCACAGCAGCCAGCGCCGCAATACAAGCCATCCACATCTCCCGC	QuikChange IE1cc172-176
820	GGTGCACTGCAGGCTAAGGCCCGTGCTGCAGCGGCTGCACTTAGGAGAAAGATGATGTATA	QuikChange IE1cc196-199
821	TATACATCATCTTTCTCCTAAGTGCAGCCGCTGCAGCACGGGCCTTAGCCTGCAGTGCACC	QuikChange IE1cc196-199
846	AAGATGATGTATATGTGCTACGCGAATATAGCGTTCTTTACCGCGAACTCAGCCTTCCCTAAGAC	QuikChange IE1cc210-217
847	GTCTTAGGGAAGGCTGAGTTCGCGGTAAAGAACGCTATATTCGCGTAGCACATATACATCATCTT	QuikChange IE1cc210-217
848	CCTCAGTGCTCCCCTGCTGCGATTATGGCTTATGCC	QuikChange IE1cc244-245
849	GGCATAAGCCATAATCGCAGCAGGGGAGCACTGAGG	QuikChange IE1cc244-245
822	GATTATGGCTTATGCCCAGAAAATATTTAAGATTTTGGCTGCGGCGGCAGCCGCGGTGCTCACGCACATTGATCACATATTTA	QuikChange IE1cc258-263
823	TAAATATGTGATCAATGTGCGTGAGCACCGCGGCTGCCGCCGCAGCCAAAATCTTAAATATTTTCTGGGCATAAGCCATAATC	QuikChange IE1cc258-263
850	GTGTGGAAACAATGTGTAATGCGTACGCGGTCACTAGTGACGCTTGTAT	QuikChange IE1cc286-292
851	ATACAAGCGTCACTAGTGACCGCGTACGCATTACACATTGTTTCCACAC	QuikChange IE1cc286-292
852	GTACAAGGTCACTAGTGCCGCTTGTATGATGACCA	QuikChange IE1cc286-292
853	TGGTCATCATACAAGCGGCACTAGTGACCTTGTAC	QuikChange IE1cc286-292
854	CATCTCTCTCTTAAGTGCGTTCTGTGCGGTGCTGTGCTGCTATG	QuikChange IE1cc307-310
855	CATAGCAGCACAGCACCGCACAGAACGCACTTAAGAGAGAGATG	QuikChange IE1cc307-310
856	GTGCTGCTATGTCTTAGCGGCGACTAGTGTGATGCTGG	QuikChange IE1cc318-319
857	CCAGCATCACACTAGTCGCCGCTAAGACATAGCAGCAC	QuikChange IE1cc318-319
858	ACTAGTGTGATGCTGGCCGCGGCGCCTCTGATAACCAAGCC	QuikChange IE1cc326-327
859	GGCTTGGTTATCAGAGGCGCCGCGGCCAGCATCACACTAGT	QuikChange IE1cc326-327
860	CAAGCGGCCTCTGATAACCGCGCCTGCGGTTATCAGTGTAATGAAG	QuikChange IE1cc332-334
861	CTTCATTACACTGATAACCGCAGGCGCGGTTATCAGAGGCCGCTTG	QuikChange IE1cc332-334
824	CAAGCCTGAGGTTATCAGTGTAATGGCGGCCGCCATTGAGGAGATCTGCATGAAGGTC	QuikChange IE1cc340-342
825	GACCTTCATGCAGATCTCCTCAATGGCGGCCGCCATTACACTGATAACCTCAGGCTTG	QuikChange IE1cc340-342
862	CATTCTGGGGGCCGCTCCTCTGGCAGTCTGCTCTCCTA	QuikChange IE1cc359-362
863	TAGGAGAGCAGACTGCCAGAGGAGCGGCCCCCAGAATG	QuikChange IE1cc359-362
826	CCATCGCCGAGGAGTCAGCTGCGGCAGCGGCTATTGTAGCCTACAC	QuikChange IE1cc379-382
827	GTGTAGGCTACAATAGCCGCTGCCGCAGCTGACTCCTCGGCGATGG	QuikChange IE1cc379-382
828	AAGAAAGTGAGCAGAGTGCTGCGGCAGCGGCGGCGGGTGCTCAGGAGGAGCG	QuikChange IE1cc432-437
829	CGCTCCTCCTGAGCACCCGCCGCCGCTGCCGCAGCACTCTGCTCACTTTCTT	QuikChange IE1cc432-437
830	GAGGAAGTTGCCCCAGCGGCAGCGGCGGCTGGTGCTGAGGAACCC	QuikChange IE1cc463-467
831	GGGTTCCTCAGCACCAGCCGCCGCTGCCGCTGGGGCAACTTCCTC	QuikChange IE1cc463-467
864	CCGCCTCTGGAGGCGCGAGCACCGCCCCTATGGTGACTA	QuikChange IE1cc478-481
865	TAGTCACCATAGGGGCGGTGCTCGCGCCTCCAGAGGCGG	QuikChange IE1cc478-481
866	GAGCACCCACCCTATGGTGACTGCAAGCGCGGCTGCCCAGTAAGAATTCTGC	QuikChange IE1cc486-490
867	GCAGAATTCTTACTGGGCAGCCGCGCTTGCAGTCACCATAGGGTGGGTGCTC	QuikChange IE1cc486-490
213	GCTTGTATTAAGGAGCCGCATGATGTGAGCAAG	QuikChange IE1 L174P
214	CTTGCTCACATCATGCGGCTCCTTAATACAAGC	QuikChange IE1 L174P
1037	CTCCTCGAAAGGCTCATGAACCTTATCTCCGCCATCTCCGGCATTCTGCAAACATCCTCCCATCATA	QuikChange IE1 L130G/I132G/L133G
1038	TATGATGGGAGGATGTTTGCAGAATGCCGGAGATGGCGGAGATAAGGTTCATGAGCCTTTCGAGGAG	QuikChange IE1 L130G/I132G/L133G
1039	GTTCTGTCGGGTGCTGTGCTGCGGTGGCGGAGAGGAGACTAGTGTGATGCTG	QuikChange IE1 Y315G/V316G/L317G
1040	CAGCATCACACTAGTCTCCTCTCCGCCACCGCAGCACAGCACCCGACAGAAC	QuikChange IE1 Y315G/V316G/L317G
320	GTGTCTGTCCGGTCTGAGCCAGTGTCTGAGATAG	QuikChange IE1 K450R
321	TGGCTCAGACCGGACAGACACAGTGTCCTCCCGC	QuikChange IE1 K450R
809	GATACTAAGCTTGCCACCATGTATCCTTACGACGTGCCTGACTACGCCGAGTCCTCTGCCAAGAGAAAGATG	PCR cloning HA-IE2
918	GATACTGAATTCTTACTGAGACTTGTTCCTCAGGTC	PCR cloning HA-IE2
1175	GATACTGAATTCGCCACCATGTATCCTTACGACGTGCCTGACTACGCCGAGTCCTCTGCCAAGAGAAAGATG	PCR cloning HA-IE1
1176	GATACTGGATCCTTACTGGTCAGCCTTGCTTCTAGT	PCR cloning HA-IE1
1177	GATACTGGATCCTTAAGAGGCGGTGGGTTCCTCAGCACC	PCR cloning HA-IE1dl476-491
701	CAGAGCTCTCCCTATCAGT	Sequencing pCMV.TetO
1046	GTGGTATGGCTGATTATGATC	Sequencing pCMV.TetO
1225	ATGTAAACCAGGGCGCCTAT	Sequencing pLVX-TetOne-Puro
1224	CCTCCTGTCTTAGGTTAGTG	Sequencing pLVX-TetOne-Puro
527	TGGCAGAACTCGGTAAGTCTGTTGACATGTATGTGATATATACTCTATATTATACTCTATAGGATGACGACGATAAGTAGGG	*En passant* mutagenesis gTBdlIE1
528	GTAGGATTACAGAGTATAACATAGAGTATAATATAGAGTATATATCACATACATGTCAACCAACCAATTAACCAATTCTGATTAG	*En passant* mutagenesis gTBdlIE1
1115	GATAAGCGGGAGATGTGGATGGCTTGTATTGCGGCGCTGGCTGCTGTGACCAAGGGCGCCGCTATAGGGATAACAGGGTAATCGATTT	*En passant* mutagenesis (g)TBIE1cc172-176
1116	CCTAACTTGTTAGCGGCGCCCTTGGTCACAGCAGCCAGCGCCGCAATACAAGCCATCCACATCTGCCAGTGTTACAACCAATTAACC	*En passant* mutagenesis (g)TBIE1cc172-176
1117	GATAAGCGGGAGATGTGGATGGCTTGTATTAAGGAGCTGCATGATGTGACCAAGGGCGCCGCTATAGGGATAACAGGGTAATCGATTT	*En passant* mutagenesis gTBrvIE1cc172-176
1118	CCTAACTTGTTAGCGGCGCCCTTGGTCACATCATGCAGCTCCTTAATACAAGCCATCCACATCTGCCAGTGTTACAACCAATTAACC	*En passant* mutagenesis gTBrvIE1cc172-176
740	GGAGCTAGAACGATTCGCAGTTA	qPCR HIV-1 Gag
739	GGTTGTAGCTGTCCCAGTATTTGTC	qPCR HIV-1 Gag
759	CAGCGAAGTGAGTTCAATGG	qPCR RPPH1
765	AATGGGCGGAGGAGAGTAGT	qPCR RPPH1
872	GCGTTTAATGTCGTCGCTCAA	qPCR hCMV UL86
873	CAGCCTACCCGTACCTTTCCA	qPCR hCMV UL86
471	TCCCTAAGACCACCAATG	RT-qPCR hCMV IE1
472	GAGCACTGAGGCAAGTTC	RT-qPCR hCMV IE1
363	TATCAGCAGTACCAGGATGC	RT-qPCR TUBB
364	TGAGAAGCCTGAGGTGATG	RT-qPCR TUBB
533	TCCACGTGTTGAGATCATTGC	RT-qPCR CXCL10
534	TCTTGATGGCCTTCGATTCTG	RT-qPCR CXCL10
688	CTGGCGGCTATAAACCTAACC	RT-qPCR OAS1
689	GTTCTGTGAAGCAGGTGGAGA	RT-qPCR OAS1
749	GGCCACTCTTCAGCATCTC	RT-qPCR SOCS3
750	ATCGTACTGGTCCAGGAACTC	RT-qPCR SOCS3
113	GACATCCCTGAGGAGATTAAG	RT-qPCR IFNB1
114	ATGTTCTGGAGCATCTCATAG	RT-qPCR IFNB1
1398	ACATTGGCAGGTTCAAATCTC	RT-qPCR IFNL1
1399	TGAGTGACTCTTCCAAGGC	RT-qPCR IFNL1
1603	TATTCCTCGGACACCACAC	RT-qPCR CCL5
1604	GTGACAAAGACGACTGCTG	RT-qPCR CCL5
1601	GAAAGCATGATCCGGGACGTG	RT-qPCR TNF
1602	GATGGCAGAGAGGAGGTTGAC	RT-qPCR TNF
111	GCTATGCATGGACCTCTG	RT-qPCR PML
112	ATGGTGGCTTGAATCTCAG	RT-qPCR PML

Plasmid pCMV-PML encoding human PML isoform VI under the control of the CMV major immediate-early promoter-enhancer has been described [125]. Plasmid template pLAY2 used for generation of mutant and revertant hCMV TB40/E BACs by *en passant* mutagenesis [126] was kindly provided by Karsten Tischer (Freie Universität Berlin).

### Antibodies

The following primary antibodies were used in this study: rabbit anti-GAPDH (Abcam, ab9485), mouse anti-HA clone 16B12 (Covance, MMS-101P), rat anti-HA clone 3F10 (Roche, 11867423001), mouse anti-histone H3 (Diagenode, C15200011-10), mouse anti-IE1 clone 1B12 [122], rabbit anti-PML (Abcam, ab72137), rabbit anti-Sp100 (Chemicon, Ab1380 for immunofluorescence and GeneTex, GTX131569 for immunoblotting), rabbit anti-SUMO1 (Epitomics, 1563–1), rabbit anti-SUMO2 (Zymed, 51–9100) and mouse anti-TUBA clone DM1A (Cell Signaling Technology, 3873).

The following secondary antibodies were used for immunoblotting: peroxidase-conjugated goat anti-mouse immunoglobulin G (IgG) (Dianova, 115-035-166), peroxidase-conjugated goat anti-rabbit IgG (Dianova, 111-035-144), IRDye 800CW-conjugated goat anti-mouse IgG (LI-COR, 925–32210), IRDye 800CW-conjugated goat anti-rabbit IgG (LI-COR, 925–32211), IRDye 800CW-conjugated goat anti-rat IgG (LI-COR, 925–32219), IRDye 680RD-conjugated goat anti-mouse IgG (LI-COR, 925–68070) and IRDye 680RD-conjugated goat anti-rabbit IgG (LI-COR, 925–68071).

The following secondary antibodies were used for immunofluorescence: Alexa Fluor 488-conjugated goat anti-mouse IgG (Thermo Fisher, A-11001), Alexa Fluor 488-conjugated goat anti-rabbit IgG (Thermo Fisher, A-11034), Alexa Fluor 594-conjugated goat anti-rabbit IgG (Thermo Fisher, A-11037) and Alexa Fluor 594-conjugated goat anti-rat IgG (Thermo Fisher, A-11007).

### Cells and lentiviruses

MRC-5 human embryonic lung fibroblasts (American Type Culture Collection, CCL-171) were maintained in Dulbecco’s Modified Eagle’s Medium (Sigma-Aldrich, D7777) containing 4.5 g/l glucose, 3.7 g/l sodium bicarbonate, 1 mM sodium pyruvate, 10% [v/v] fetal calf serum, 100 units/ml penicillin and 100 μg/ml streptomycin. The human embryonic kidney cell line 293T (GenHunter, Q401) was cultured in the same medium in the presence of 400 μg/ml G418 sulfate. All cultures were regularly screened for *Mycoplasma sp*. using a PCR assay [127]. Production of replication-deficient lentiviral particles, lentivirus infections and selection of stable cell lines were performed as described [52, 72]. To generate luciferase and HA-IE1 expressing cells using the Lenti-X TetOne inducible expression system (Clontech), low-passage MRC-5 cells were transduced twice for 4 h with pLVX-TetOne-Puro-derived lentiviruses and selected with 1 μg/ml puromycin dihydrochloride (Sigma-Aldrich, P8833). To induce IE1 expression, cells were treated with dox (Clontech, 631311) at a final concentration of 1 μg/ml. Where indicated, cells were treated with 1,000 U/ml recombinant human IFNα A/D (R&D Systems, 11200) for 24 h.

### hCMV mutagenesis and infection

Wild-type virus of the low passage hCMV strain TB40/E (TBwt) was derived from the bacterial artificial chromosome (BAC) TB40-BAC4 [128]. A modified version of this BAC with an SV40-EGFP-BGH PolyA cassette inserted between the US34 and TRS1 genes [129] was used to generate the EGFP expressing TB40/E wild-type virus gTBwt. Mutant BACs encoding no IE1 (gTBdlIE1) or IE1 with amino acids 172, 173, 175 and 176 changed to alanine (pTBIE1cc172-176 and pgTBIE1cc172-176) and the revertant BAC pgTBrvIE1cc172-176 were generated by markerless *en passant* mutagenesis as described [130] using plasmid pLAY2 and oligonucleotide primers listed in Table 1. The identity and integrity of each BAC were verified by Sanger sequencing of the modified region and restriction fragment length analysis following digestion with *Eco*RI. Viruses were reconstituted and virus stocks produced upon electroporation of MRC-5 cells with BAC DNA following standard protocols. Viruses (g)TBwt, (g)TBIE1cc172-176 and gTBrvIE1cc172-176 were grown on normal MRC-5 fibroblasts while gTBdlIE1 was produced on MRC-5 cells with constitutive expression of wild-type IE1 following transduction with pLKO.DCMV.TetO.cIE1-derived lentivirus [72].

Titers of wild-type and revertant virus preparations were determined by plaque assay on MRC-5 cells in 6-well plates. For plaque assays, infections were performed in triplicates for 16 h with 800 μl inoculum before cells were overlayed with 4 ml Dulbecco’s Modified Eagle’s Medium supplemented with 3.7 g/l sodium hydrogen carbonate, 1% [w/v] methyl cellulose (Sigma-Aldrich, M0262), 2% [v/v] fetal calf serum, 100 units/ml penicillin and 100 μg/ml streptomycin. Plaques of EGFP-positive viruses were counted at day 9 post infection using an EVOS FL Cell Imaging System (Thermo Fisher) with Plan Achromat 4× Objective and EVOS Light Cube for GFP. Foci of three or more fluorescent cells were counted as plaques. Plaques formed by EGFP-negative viruses were counted at day 12 post infection in phase contrast mode after staining of cell monolayers with 0.5% [w/v] methylene blue in 70% [v/v] methanol. For quantification of infectious viral genome equivalents, 5 × 10^5^ MRC-5 cells were seeded onto 6-well dishes and infected 40 h later in triplicates with 800 μl 1:10-diluted virus stock in growth medium supplemented with 50 U/ml benzonase nuclease (Sigma-Aldrich, E1014) to remove free viral DNA. Plates were incubated for 1 h at 37°C with occasional rocking before the virus inoculum was removed and cells were washed in 5 ml phosphate-buffered saline (PBS) with 10 mM ethylenediaminetetraacetic acid (EDTA). Cells were dislodged and extracellular virus was removed by treatment with 500 μl trypsin (0.5 g/l) and EDTA (0.5 mM) solution for 5 min at 37°C. Cells were transferred to 2-ml tubes using 1.3 ml PBS/EDTA and collected by centrifugation for 5 min at 800 g. Cell pellets were subjected to an additional washing step with 1.8 ml PBS/EDTA before the supernatant was removed and cells were resuspended in 200 μl PBS/EDTA and transferred to 1.5-ml tubes for DNA isolation and qPCR analysis.

To monitor replication of gTBdlIE1 on trans-complementing cell lines, confluent MRC-5 cultures on 12-well dishes were infected at the indicated MOI by applying 300 μl virus dilution supplemented with dox. At 16 h (day 1) after infection, the inoculum was removed, cells were washed twice with 2 ml growth medium and further incubated in 1 ml growth medium with dox. On day 2 post infection and every other day thereafter, half of the growth medium was replaced and viral replication was assessed by qPCR-based relative quantification of hCMV DNA from culture supernatants. To compare replication of wild-type, mutant and revertant gTB viruses, 2.5 × 10^4^ MRC-5 cells were seeded in a volume of 100 μl onto 96-well plates and infected 24 h later at the indicated MOI by adding 100 μl virus dilution. EGFP fluorescence was measured in a Tecan Infinite M200 PRO microplate reader immediately after virus addition and at day 4, 8, 12 and 16 post infection. The following instrument settings were used: measurement mode, fluorescence intensity bottom; excitation wavelength, 483 nm; excitation bandwidth, 9 nm; emission wavelength, 535 nm; emission bandwidth, 20 nm; gain, 80; integration time, 20 μs; flashes, 4 × 7. The signal at day 0 was considered background and was subtracted from all other measurements. For RNA analyses, 2.5 × 10^5^ MRC-5 cells were seeded onto 12-well dishes and infected 72 h later at the indicated MOI by applying 300 μl virus dilution. After 2 h, 700 μl pre-warmed growth medium was added and the cells were harvested at the indicated times post infection. For immunocytochemistry, 5 × 10^5^ MRC-5 cells were seeded in 6-well dishes and infected 16 h later at the indicated MOI by applying 800 μl virus dilution. After 2 h, 2 ml 37°C growth medium was added and cells were fixed at the indicated times post infection.

### DNA and RNA analyses

To determine steady-state mRNA levels by RT-qPCR, total RNA was isolated from fibroblasts cultured in 12-well dishes using the RNeasy Mini Plus Kit according to Qiagen’s instructions. First-strand cDNA was synthesized at 50°C using the AffinityScript Multiple Temperature cDNA Synthesis Kit and oligo(dT) primers (Agilent Technologies). First-strand cDNA was diluted 10-fold in sterile ultrapure water, and 5 μl was used for real-time PCR as described in detail in previous publications [52, 124]. The sequences of oligonucleotide primers used for RT-qPCR are shown in Table 1.

For viral genome quantification, total DNA was extracted from cells grown in 6-well dishes or from 200 μl pre-spun (4000 g, 10 min) culture supernatant using a DNeasy Blood & Tissue Kit (Qiagen) according to the manufacturer’s spin-column protocol for animal blood and cells. DNA was released from the column in two consecutive steps with 100 μl (supernatant samples) or 200 μl (cell samples) elution buffer. DNA from infected cells was diluted 10-fold, and 5-μl samples were subjected to real-time PCR with primers shown in Table 1.

### Protein analyses

For indirect immunofluorescence microscopy, cells were grown on precision cover glasses (Marienfeld Superior, No. 1.5H), fixed with pre-chilled methanol for 20 min at -20°C and processed as described [52, 72].

Whole cell lysates were prepared in 10 mM PIPES-NaOH, pH 7.2 or 50 mM Tris-HCl, pH 8.0 buffer with 150 mM NaCl, 0.1% [w/v] sodium dodecyl sulfate (SDS), 1% [v/v] Igepal CA-630, 0.5% [w/v] sodium deoxycholate and 1% [v/v] protease inhibitor cocktail (Merck, 539134). Where indicated, cell lysis buffer was supplemented with 20 mM iodoacetamide (IAA) and 20 mM N-ethylmaleimide (NEM) from freshly prepared 0.5 M IAA and 1 M NEM stock solutions in water and methanol, respectively. Cell extracts were combined with an equal volume of 2 × protein sample buffer (100 mM Tris-HCl, pH 6.8, 4% [w/v], SDS, 20% [v/v] glycerol, 0.2% [w/v] orange G, 200 mM beta-mercaptoethanol), heated for 5 min at 95°C, cooled on ice and sonicated in a Bioruptor UCD-200 (Diagenode) in high intensity mode for 15 min (30 sec ON/30 sec OFF) to maximize protein solubilization and shear chromatin. Insoluble material was removed by centrifugation at 20,000 × *g*, 4°C for 10 min, and equal sample volumes were used for SDS polyacrylamide gel electrophoresis and immunoblotting as described [45, 52, 55, 124].

For co-immunoprecipitation experiments, 5 x 10^6^ 293T cells were seeded onto 10-cm dishes and transfected 24 h later with 10 μg pCMV-PML and 10 μg pLKO.DCMV.TetO IE1 expression plasmid or empty vector using the calcium-phosphate co-precipitation technique [131]. At 48 h post transfection, cells were cross-linked by treatment with 1% [v/v] formaldehyde for 10 min at room temperature. To stop cross-linking, glycine was added to a final concentration of 125 mM, and samples were incubated for another 5 min at room temperature. Cells were washed and harvested with 10 ml ice-cold PBS and lysed in 500 μl 10 mM PIPES-NaOH, pH 7.2 buffer with 150 mM NaCl, 0.1% [w/v] sodium dodecyl sulfate (SDS), 1% [v/v] Igepal CA-630, 0.5% [w/v] sodium deoxycholate, 1% [v/v] protease inhibitor cocktail and 20 mM NEM for 15 min on ice. Extracts were sonicated in a Bioruptor UCD-200 in high intensity mode for 15 min (30 sec ON/30 sec OFF) and cleared by centrifugation at 20,000 × *g*, 4°C for 30 min. The supernatant (475 μl) was combined with 20 μl pre-washed anti-HA magnetic bead slurry (Thermo Fisher, 88837) and incubated for 2 h at 4°C with gentle rotation. Immune complexes were washed with 1 ml cell lysis and 1 ml nuclease reaction buffer (50 mM Tris-HCl pH 8.0, 2 mM MgCl_2_) before incubation with 25 U benzonase nuclease in 100 μl nuclease reaction buffer for 30 min at 4°C. After four additional washing steps with 1 ml cell lysis buffer, proteins were eluted by addition of 30 μl 1× protein sample buffer and incubation for 10 min at 99°C.

For SUMOylation analysis, about 5×10^6^ MRC-5 cells were lysed for 15 min on ice in 600 μl 50 mM Tris-HCl, pH 8.0 buffer containing 150 mM NaCl, 2 mM MgCl_2_, 1% [v/v] Triton X-100, 1% [v/v] protease inhibitor cocktail, 2% [v/v] phosphatase inhibitor cocktail II (Merck, 524625), 20 mM IAA, 20 mM NEM, and 25 U/ml benzonase nuclease. After sonification in a Bioruptor Pico (Diagenode) for three cycles (30 sec ON/30 sec OFF), EDTA was added to a final concentration of 20 mM and lysates were cleared by centrifugation at 20,000 × *g*, 4°C for 10 min. From the supernatant, 500 μl was added to 20 μl pre-washed anti-HA magnetic beads, and samples were incubated for 90 min at 4°C with gentle rotation. Immune complexes were washed four times with 1 ml 50 mM Tris-HCl, pH 8.0 buffer containing 150 mM NaCl, 1 mM EDTA, 1% [v/v] Triton X-100, 1% [v/v] protease inhibitor cocktail, 20 mM IAA and 20 mM NEM, and proteins were eluted by addition of 60 μl 2× protein sample buffer and incubation for 5 min at 95°C.

For subnuclear fractionation, approximately 5×10^6^ MRC-5 cells were gently resuspended in 200 μl freshly prepared CSK buffer (10 mM PIPES-NaOH, pH 6.8, 100 mM NaCl, 300 mM sucrose, 3 mM MgCl_2_, 1% [v/v] protease inhibitor cocktail, 20 mM IAA, 20 mM NEM) with 0.1% [v/v] Igepal CA-630. Nuclei were spun down at 1,300 × *g*, 4°C for 1 min, and the supernatant was collected as cytoplasm fraction. The pellet was washed with 300 μl detergent-free CSK buffer before the nucleoplasm was isolated with 200 μl CSK buffer containing 0.5% [v/v] Triton X-100. The nuclear pellet remaining after another 1-min spinning step at 1,300 × *g*, 4°C was washed with 300 μl 50 mM Tris-HCl, pH 7.5, 5 mM CaCl_2_ and resuspended in 200 μl of the same buffer containing 100 units micrococcal nuclease (Thermo Fisher, 88216) to solubilize chromatin. Samples were sonicated for 10 sec in a Bioruptor Pico to improve the accessibility of chromatin for the enzyme before tubes were incubated for 10 min at 30°C with shaking. After that, samples were supplemented with 0.5% [v/v] Triton X-100 and sonicated again for 3 min (30 sec ON/30 sec OFF). The soluble chromatin fraction was removed after spinning at 10,000 × *g*, 4°C for 5 min, and the insoluble pellet material was washed once with 200 μl CSK buffer containing 0.5% [v/v] Triton X-100 before it was resuspended as matrix fraction in 200 μl 2× protein sample buffer.

### Protein modeling and structure visualization

The Normal Modelling Mode provided with the Protein Homology/analogY Recognition Engine 2.0 (Phyre2) [132] was used to model the three-dimensional structure of the hCMV (Towne) IE1 protein (GenBank AAR31448). 70% of the sequence, corresponding to the IE1 core domain (amino acids 25 to 378), were modelled with 100% confidence by RhCMV IE1/rhUL123 (PDB 4WID) [49], the single highest scoring template. Protein structures were rendered and annotated using UCSF Chimera 1.13.1 (Resource for Biocomputing, Visualization, and Informatics) [133].

## Supporting information

S1 Fig Subcellular localization and disruption of PML bodies by wild-type and mutant IE1. (A) TetR (w/o), TetR-IE2 and TetR-IE1 cells expressing the indicated HA-tagged wild-type (wt) or mutant IE1 proteins were treated with dox for 24 h. Indirect immunofluorescence staining was performed using mouse anti-HA and rabbit anti-PML combined with goat anti-mouse Alexa Fluor 488 and goat anti-rabbit Alexa Fluor 594 antibodies. DAPI was used to stain DNA. Representative merge images from cells showing the localization of IE1, IE2 and PML relative to DNA are presented (Keyence BZ-9000 microscope, 40× objective). The percentage of cells exhibiting predominantly disrupted or intact PML bodies is shown in Fig 3C.
(TIF)

S2 Fig Regulation of STAT signaling by wild-type IE1 and clustered charge mutants defective in PML targeting. TetR (w/o) and TetR-IE1 cells expressing the indicated wild-type (wt) or clustered charge mutant IE1 proteins were treated with dox for 96 h and solvent or IFNα for 24 h. Relative mRNA levels were determined by RT-qPCR for typical STAT1- (CXCL10), STAT2- (OAS1) and STAT3- (SOCS3) responsive genes and normalized to TUBB. For CXCL10 and SOCS3, results from solvent-treated cells are shown relative to cells without IE1 (set to 1). For OAS1, the fold increase in the presence of IFNα was calculated, and results are presented relative to wt cells (set to 1).
(EPS)

S3 Fig Regulation of STAT signaling and interaction with PML bodies by wild-type and mutant IE1 proteins. Growth-arrested TetR (w/o) and TetR-IE1 cells expressing the indicated wild-type (wt) or mutant IE1 proteins were treated with dox for 72 h. (A) Relative mRNA levels were determined by RT-qPCR for IE1 and typical STAT1- and STAT3-responsive genes (CXCL10 and SOCS3, respectively) and normalized to TUBB. Data presented are means and standard deviations of two biological and two technical replicates. (B) Cells were treated with IFNα for 24 h. Relative mRNA levels were determined by RT-qPCR for IE1 and a typical STAT2-responsive gene (OAS1) and normalized to TUBB. Data presented are means and standard deviations of two biological and two technical replicates. (C) Indirect immunofluorescence staining was performed using mouse anti-IE1 and rabbit anti-PML combined with goat anti-mouse Alexa Fluor 594 and goat anti-rabbit Alexa Fluor 488 antibodies. DAPI was used to stain DNA. Individual and merge images were taken using a Keyence BZ-9000 microscope (40× objective).
(EPS)

S4 Fig Metabolic stability of wild-type and mutant IE1 proteins. TetOne-IE1 cells with tightly controlled inducible expression of the indicated HA-tagged wild-type (wt) or mutant IE1 proteins were treated with dox (1 μg/ml) for 12 h. Cells were then either collected (0 h) or washed three times with prewarmed growth medium and incubated for another 12, 24, 36, 48 or 60 h in the absence of dox. Whole cell protein extracts were prepared and analyzed by quantitative immunoblotting using rat anti-HA and mouse anti-TUBA combined with goat anti-rat IRDye 800CW (green) and goat anti-mouse IRDye 680RD (red) antibodies.
(EPS)

S5 Fig Disruption of PML bodies in cells infected with IE1cc172-176 mutant hCMV. (A) MRC-5 cells were infected with gTBdlIE1, gTBwt, gTBIE1cc172-176 or gTBIE1rv172-176 at an MOI of 2 PFUs/cell for 16 h. Indirect immunofluorescence staining was performed using mouse anti-IE1 and rabbit anti-PML combined with goat anti-mouse Alexa Fluor 488 and goat anti-rabbit Alexa Fluor 594 antibodies. DAPI was used to stain DNA. Individual and merge images were taken using a Keyence BZ-9000 microscope (40× objective). (B) MRC-5 cells were infected with TBdlIE1, TBwt or TBIE1cc172-176 at an MOI of 2 PFUs/cell for 16 h. Indirect immunofluorescence staining was performed using mouse anti-IE1 and rabbit anti-PML combined with goat anti-mouse Alexa Fluor 488 and goat anti-rabbit Alexa Fluor 594 antibodies. DAPI was used to stain DNA. Images were taken using a Keyence BZ-9000 microscope (40× objective). (C) The percentage of nuclei exhibiting predominantly disrupted or undisrupted PML bodies was determined for at least 100 cells using images acquired as described in (B).
(TIF)

S6 Fig Replication of IE1cc172-176 mutant compared to wild-type and revertant hCMV. MRC-5 cells were infected with gTBwt, gTBIE1cc172-176 or gTBrvIE1cc172-176 at an MOI of 0.5 PFU/cell. Every 48 h, half of the culture medium was replaced and viral replication was assessed by qPCR-based relative quantification of hCMV DNA from culture supernatants with primers specific for UL86. Data presented are means and standard deviations of three biological and two technical replicates.
(EPS)
