# Supplementary figures and images for: Revisiting promyelocytic leukemia protein targeting by human cytomegalovirus immediate-early protein 1
Source: PLoS Pathog. 2020 May 4;16(5):e1008537. doi: 10.1371/journal.ppat.1008537 (PMC7224577; doi:10.1371/journal.ppat.1008537)

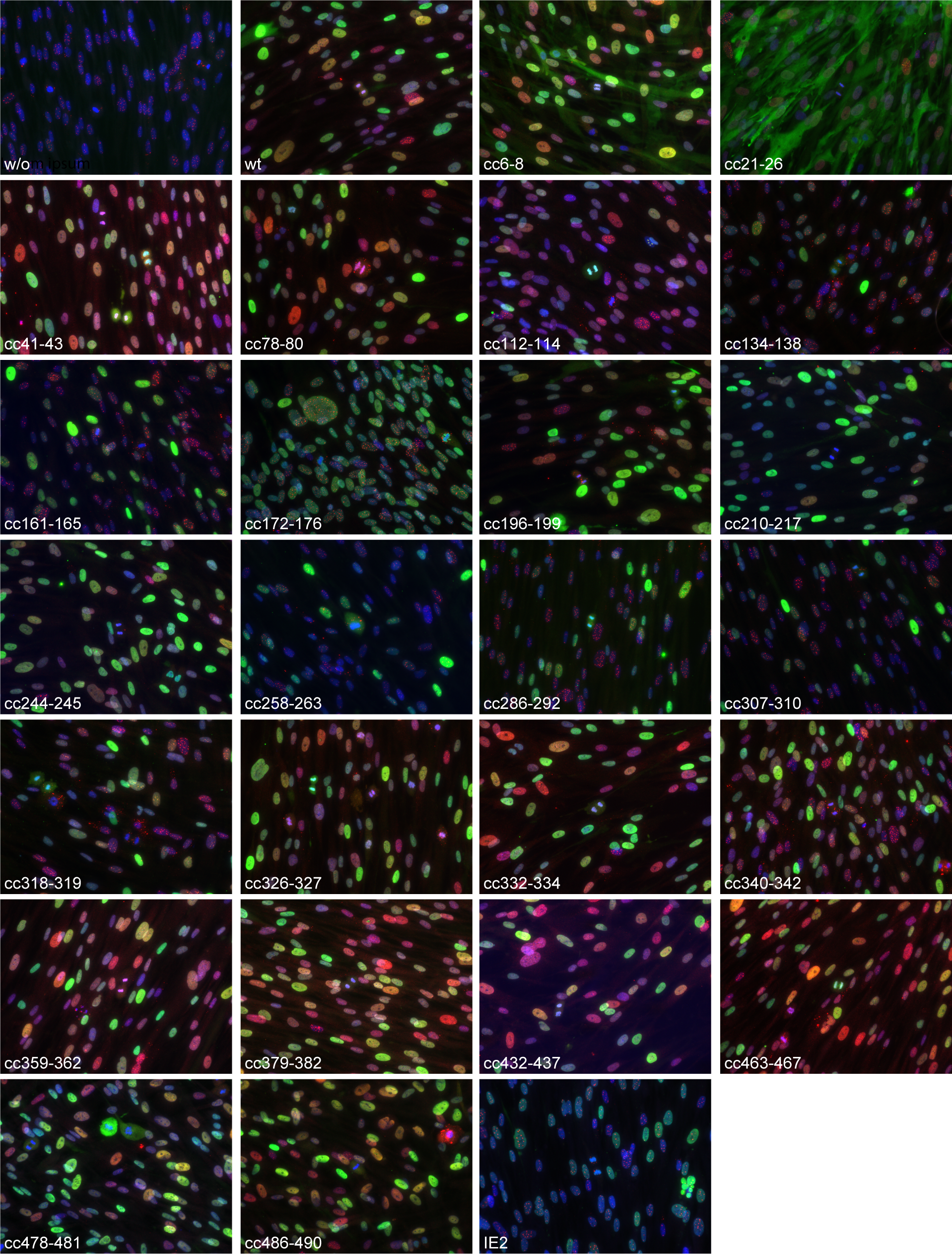

Supplement: S1 Fig — (A) TetR (w/o), TetR-IE2 and TetR-IE1 cells expressing the indicated HA-tagged wild-type (wt) or mutant IE1 proteins were treated with dox for 24 h. Indirect immunofluorescence staining was performed using mouse anti-HA and rabbit anti-PML combined with goat anti-mouse Alexa Fluor 488 and goat anti-rabbit Alexa Fluor 594 antibodies. DAPI was used to stain DNA. Representative merge images from cells showing the localization of IE1, IE2 and PML relative to DNA are presented (Keyence BZ-9000 microscope, 40× objective). The percentage of cells exhibiting predominantly disrupted or intact PML bodies is shown in Fig 3C. (TIF) [file ppat.1008537.s001.tif]

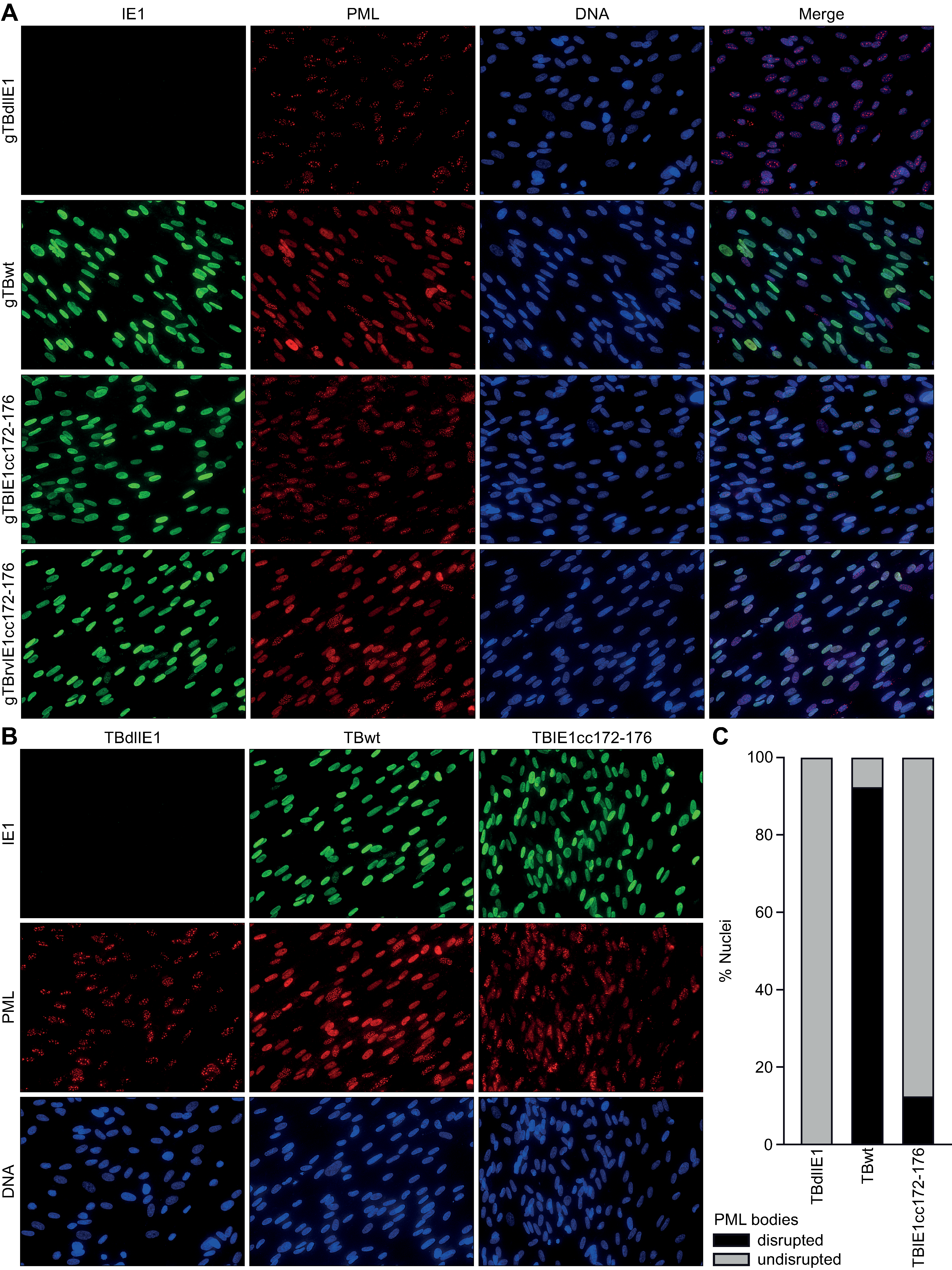

Supplement: S5 Fig — (A) MRC-5 cells were infected with gTBdlIE1, gTBwt, gTBIE1cc172-176 or gTBIE1rv172-176 at an MOI of 2 PFUs/cell for 16 h. Indirect immunofluorescence staining was performed using mouse anti-IE1 and rabbit anti-PML combined with goat anti-mouse Alexa Fluor 488 and goat anti-rabbit Alexa Fluor 594 antibodies. DAPI was used to stain DNA. Individual and merge images were taken using a Keyence BZ-9000 microscope (40× objective). (B) MRC-5 cells were infected with TBdlIE1, TBwt or TBIE1cc172-176 at an MOI of 2 PFUs/cell for 16 h. Indirect immunofluorescence staining was performed using mouse anti-IE1 and rabbit anti-PML combined with goat anti-mouse Alexa Fluor 488 and goat anti-rabbit Alexa Fluor 594 antibodies. DAPI was used to stain DNA. Images were taken using a Keyence BZ-9000 microscope (40× objective). (C) The percentage of nuclei exhibiting predominantly disrupted or undisrupted PML bodies was determined for at least 100 cells using images acquired as described in (B). (TIF) [file ppat.1008537.s005.tif]
